# Addressing global disparities in blood pressure control: perspectives of the International Society of Hypertension

**DOI:** 10.1093/cvr/cvac130

**Published:** 2022-10-11

**Authors:** Aletta E Schutte, Tazeen H Jafar, Neil R Poulter, Albertino Damasceno, Nadia A Khan, Peter M Nilsson, Jafar Alsaid, Dinesh Neupane, Kazuomi Kario, Hind Beheiry, Sofie Brouwers, Dylan Burger, Fadi J Charchar, Myeong-Chan Cho, Tomasz J Guzik, Ghazi F Haji Al-Saedi, Muhammad Ishaq, Hiroshi Itoh, Erika S W Jones, Taskeen Khan, Yoshihiro Kokubo, Praew Kotruchin, Elizabeth Muxfeldt, Augustine Odili, Mansi Patil, Udaya Ralapanawa, Cesar A Romero, Markus P Schlaich, Abdulla Shehab, Ching Siew Mooi, U Muscha Steckelings, George Stergiou, Rhian M Touyz, Thomas Unger, Richard D Wainford, Ji-Guang Wang, Bryan Williams, Brandi M Wynne, Maciej Tomaszewski

**Affiliations:** School of Population Health, University of New South Wales, Kensington Campus, High Street, Sydney 2052 NSW, Australia; The George Institute for Global Health, King Street, Newton, Sydney NSW 2052, Australia; Hypertension in Africa Research Team, SAMRC Unit for Hypertension and Cardiovascular Disease; North-West University, Hoffman Street, Potchefstroom 2520, South Africa; SAMRC Development Pathways for Health Research Unit, School of Clinical Medicine, University of the Witwatersrand, 1 Jan Smuts Ave, Braamfontein, Johannesburg, 2000, South Africa; Program in Health Services and Systems Research, Duke-NUS Medical School, Department of Renal Medicine, 8 College Rd., Singapore 169857, Singapore; Duke Global Health Institute, Duke University, 310 Trent Dr, Durham, NC 27710, USA; Imperial Clinical Trials Unit, School of Public Health, Imperial College London, London W12 7RH, UK; Faculty of Medicine, Eduardo Mondlane University, 3453 Avenida Julius Nyerere, Maputo, Mozambique; Department of Medicine, University of British Columbia, Vancouver, British Columbia, Canada; Center for Health Evaluation and Outcomes Sciences, Vancouver, British Columbia, Canada; Department of Clinical Sciences, Skane University Hospital, Lund University, Malmö, Sweden; Ochsner Health System, New Orleans, Louisiana, USA; Queensland University, Brisbane, Queensland, Australia; Department of International Health, Johns Hopkins Bloomberg School of Public Health, Johns Hopkins University, Baltimore, MD, USA; Division of Cardiovascular Medicine, Department of Medicine, Jichi Medical University School of Medicine, Tochigi, Japan; International University of Africa, Khartoum, Sudan; Department of Cardiology, Cardiovascular Center Aalst, OLV Clinic Aalst, Aalst, Belgium; Department of Experimental Pharmacology, Faculty of Medicine and Pharmacy, Vrije Universiteit Brussel, Brussels, Belgium; Kidney Research Centre, Ottawa Hospital Research Institute, Department of Cellular and Molecular Medicine, University of Ottawa, Ottawa, Ontario, Canada; Health Innovation and Transformation Centre, Federation University, Ballarat, Victoria, Australia; Department of Physiology and Anatomy, University of Melbourne, Melbourne, Victoria, Australia; Department of Internal Medicine, College of Medicine, Chungbuk National University, Cheongju, Korea; Institute of Cardiovascular and Medical Sciences, University of Glasgow, Glasgow, UK; Baghdad College of Medicine, Baghdad, Iraq; Pakistan Hypertension League, Karachi, Pakistan; Department of Endocrinology, Metabolism and Nephrology, School of Medicine, Keio University, 35 Shinanomachi, Shinjuku-ku, Tokyo 160-8585, Japan; Division of Nephrology and Hypertension, Groote Schuur Hospital and Kidney and Hypertension Research Unit, University of Cape Town, Cape Town, South Africa; Department of Public Health Medicine, University of Pretoria, Pretoria, South Africa; Department of Preventive Cardiology, National Cerebral and Cardiovascular Center, Osaka, Japan; Department of Emergency Medicine, Faculty of Medicine, Khon Kaen University, Khon Kaen, Thailand; University Hospital Clementino Fraga Filho, Hypertension Program, Universidade Federal do Rio de Janeiro, Brazil; Circulatory Health Research Laboratory, College of Health Sciences, University of Abuja, Abuja, Nigeria; Department of Nutrition and Dietetics, Asha Kiran JHC Hospital, Chinchwad, India; Faculty of Medicine, University of Peradeniya, Kandy, Central Province, Sri Lanka; Renal Division, Department of Internal Medicine, Emory University School of Medicine, Atlanta, GA, USA; Dobney Hypertension Centre, School of Medicine, Royal Perth Hospital Unit and RPH Research Foundation, The University of Western Australia, Perth, Australia; Department of Cardiology, Royal Perth Hospital, Perth, Western Australia, Australia; Department of Nephrology, Royal Perth Hospital, Perth, Western Australia, Perth, Western Australia, Australia; College of Medicine and Health Sciences, United Arab Emirates University, Al Ain, United Arab Emirates; Department of Family Medicine, Faculty of Medicine and Health Sciences, Universiti Putra Malaysia, Malaysia; Department of Cardiovascular & Renal Research, Institute of Molecular Medicine. University of Southern Denmark, Odense, Denmark; Hypertension Centre STRIDE-7, School of Medicine, Third Department of Medicine, Sotiria Hospital, National and Kapodistrian University of Athens, Athens, Greece; Research Institute of the McGill University Health Centre, McGill University, Montreal, QC, Canada; CARIM - Cardiovascular Research Institute, Maastricht University, Maastricht, The Netherlands; Department of Pharmacology & Experimental Therapeutics and the Whitaker, Cardiovascular Institute, Boston University School of Medicine, Boston, MA, USA; Department of Hypertension, Centre for Epidemiological Studies and Clinical Trials, The Shanghai Institute of Hypertension, Shanghai Key Laboratory of Hypertension, Ruijin Hospital, Shanghai Jiaotong University School of Medicine, Shanghai, China; Institute of Cardiovascular Science, University College London (UCL), National Institute for Health Research (NIHR), UCL Hospitals Biomedical Research Centre, London, UK; Department of Internal Medicine, Division of Nephrology & Hypertension, University of Utah, Salt Lake City, UT, USA; Division of Cardiovascular Sciences, Faculty of Medicine, Biology and Health, University of Manchester, Manchester, UK; Manchester Heart Centre, Manchester University NHS Foundation Trust, Manchester, UK; Manchester Academic Health Science Centre, Manchester University NHS Foundation Trust, Manchester, UK

**Keywords:** Epidemiology, Hypertension, Global, International, Cardiovascular disease, Regions, Inequity, Prevention, Awareness, Treatment, Control

## Abstract

Raised blood pressure (BP) is the leading cause of preventable death in the world. Yet, its global prevalence is increasing, and it remains poorly detected, treated, and controlled in both high- and low-resource settings. From the perspective of members of the International Society of Hypertension based in all regions, we reflect on the past, present, and future of hypertension care, highlighting key challenges and opportunities, which are often region-specific. We report that most countries failed to show sufficient improvements in BP control rates over the past three decades, with greater improvements mainly seen in some high-income countries, also reflected in substantial reductions in the burden of cardiovascular disease and deaths. Globally, there are significant inequities and disparities based on resources, sociodemographic environment, and race with subsequent disproportionate hypertension-related outcomes. Additional unique challenges in specific regions include conflict, wars, migration, unemployment, rapid urbanization, extremely limited funding, pollution, COVID-19-related restrictions and inequalities, obesity, and excessive salt and alcohol intake. Immediate action is needed to address suboptimal hypertension care and related disparities on a global scale. We propose a Global Hypertension Care Taskforce including multiple stakeholders and societies to identify and implement actions in reducing inequities, addressing social, commercial, and environmental determinants, and strengthening health systems implement a well-designed customized quality-of-care improvement framework.

**A video prepared by authors can also be viewed here: Supplementary material**

## 1. Introduction

Raised blood pressure (BP) is the leading attributable risk factor for death globally, accounting for 10.8 million deaths in 2019.^1^ During the past four decades, the number of people with hypertension has increased by 90%, mainly in low- and middle-income countries (LMICs). The main drivers for this rise are population growth, ageing, unhealthy environments, and behaviours (sedentary lifestyles, poor diets, obesity, alcohol abuse), and differences in access to quality care. Consequently, there are considerable disparities in age-standardized BP levels, BP control, and related cardiovascular disease (CVD) burden globally, among and within countries.^2^

**Figure 1:**
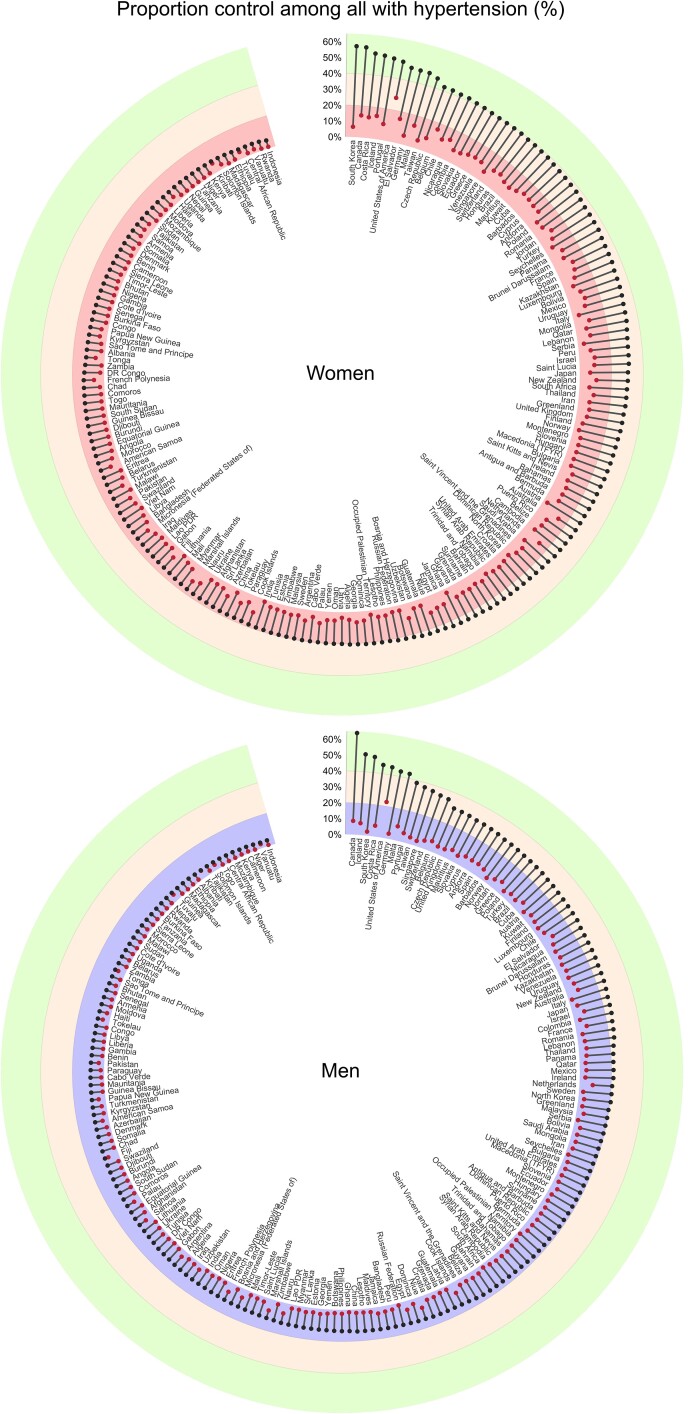
Change in absolute blood pressure control rates from 1990 to 2019 by country and sex (adapted with permission from the NCD Risk Factor Collaboration database^4,5^). Red dots (control rates 1990), black dots (control rates 2019).

**Figure 2:**
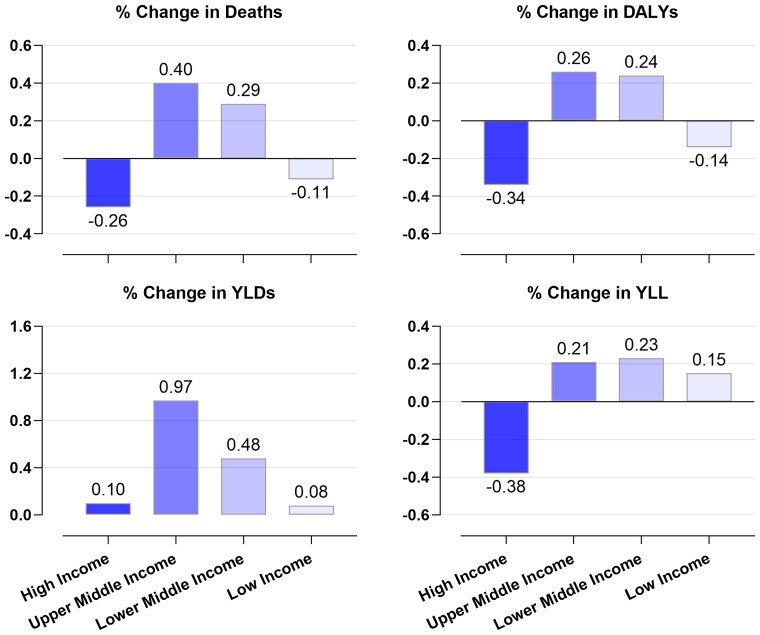
Global trends in hypertension-related CVD morbidity and mortality (1990–2019).^6^ Reproduced with permission: percentage change in deaths, disability-adjusted life-years (DALYs), years lived with disability (YLDs), and years of life lost (YLLs) due to high systolic blood pressure according to the World Bank income classification of countries between 1990 and 2019.

In most high-income countries (HICs), the observed age-standardized BP levels have declined, and hypertension awareness and treatment have improved with control rates at ∼50% (*Figure 1*).^2,3^ However, the highest BP levels have shifted from HIC to LMICs.^6,7^ BP levels rose in South and East Asia, and sub-Saharan Africa (SSA) with poor rates of awareness, treatment, and control (<10%).^2,4,8,9^ Moreover, populations in Central and Eastern Europe, the Middle East, and North Africa continue to have the highest BP levels and suboptimal BP control (∼30%) (*Figure 1*).^2,4^ Consequently, trends of hypertension-related CVD deaths declined more in HIC compared with LMICs, while the years lived with related disability increased in all countries, albeit more in LMICs (*Figure 2*).^6^ In addition, significant race-based disparities exist in BP control even within HICs, with certain subgroups more likely to be under-treated with worse BP control,^10^ and disproportionately affected by related cardiovascular complications.^11^

The NCD Risk Factor Collaboration and Global Burden of Disease Collaboration have been pivotal in providing global statistics to demonstrate the alarming situation that we now face—an asymptomatic but leading risk factor for death, amidst many other (urgent) global challenges such as COVID-19 and climate change. Major steps have already been taken to improve hypertension care globally, recognizing that drastic actions are urgently needed to turn the dial. These include the World Health Organisation (WHO)’s development of the HEARTS technical package^12^ to provide a strategic approach to improve cardiovascular health. Modules include Healthy lifestyle counselling, Evidence-based treatment protocols, Access to essential medicines and technology, Risk-based CVD management, Team-based care, Systems for monitoring, and an implementation guide. Their efforts have been supported by Resolve to Save Lives—a global public health organization that identified the urgency to prevent millions of deaths in LMICs from CVDs through speed, simplicity, and scale. The Lancet Commission on Hypertension^13^ urged for a life-course strategy by creating healthy environments, highlighted improving awareness, and actions on many elements of the WHO HEARTS package, such as workforce expansion, plus measurement and medication access (overcoming treatment inertia and improving non-adherence to medication). Despite numerous ‘calls to action’,^13–17^ limited improvement has been seen. But there are some success stories where clear improvements are evident. One example is the adoption of the HEARTS package in the Americas led by the Pan American Health Organisation. In <1 year, the proportion of those treated and who were controlled increased from 59 to 69% in Cuba.^18^

The purpose of this paper is not to reiterate all well-known barriers, but to provide region-specific evidence and often unique perspectives from the ground level. In recognition of contributions by members of the International Society of Hypertension (ISH) to hypertension research over the last few decades, *Cardiovascular Research* has extended an invitation to the President of the Society to contribute a state-of-the-art review article summarizing the past, present, and future in hypertension around the globe. A call for an expression of interest has been sent to the ISH Council, Committees and Regional Advisory Groups. Expressions were reviewed considering expertise, global representation, career stage, and gender balance. Contributors were divided into eight working groups aligned with the key tasks and ISH global structure of Regional Advisory Groups, each led by an experienced ISH investigator. Further to internal review, the ISH College of Experts selected external reviewers (including WHO, the European Society of Hypertension, and World Hypertension League). Their comments were addressed before submission to the journal. From the perspective of the ISH, this paper will thus outline:

1. Lessons learnt over the past 50 years. Key steps taken over the past decades in the management of hypertension including BP-lowering agents, the non-pharmacological management of hypertension, landmark trials, guidelines, treatment, and control.
2. The impact of raised BP worldwide. This section highlights unique region-specific challenges and opportunities based on evidence and experiences of ISH members. Regions include SSA, the Americas, Europe, the Middle East and North Africa, South and Central Asia, and the Asia Pacific;
3. Actions to ensure evidence-based hypertension care for cardiovascular risk reduction. This section provides strategies to improve hypertension care and to eliminate related disparities among and within countries.

## 2. Lessons learnt over the past 50 years

The history of BP measurement began almost three centuries ago (*Table 1*) whilst the main drug classes which dominate current clinical practice were developed between 30 and 60 years ago (*Table 2*). After their development, the newly developed agents were included in multiple clinical trials (*Table 3*), the results of which led to the development of national and international guidelines on hypertension management (*Table 4*). The initial section of this review attempts to record how the inter-relationships amongst these drug classes, trials, and guidelines relate to the evolution of clinical management of hypertension in the last five to six decades.

**Table 1:** Major steps in the evolution of BP measurement^19–21^

1733	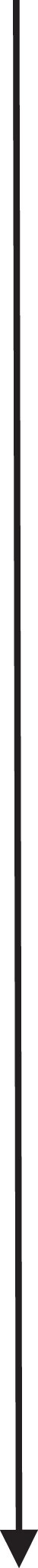	First intra-arterial BP measurement (*Stephen Hales*)
1833		First device with mercury bulb and glass column (*Jules Herisson*)
1854		First non-invasive mechanical sphygmograph (*Karl Vierordt*)
1880		Invention of sphygmomanometer (*Siegfried Ritter von Basch*)
1896		Invention of cuff (*Scipione Riva-Rocci*)
1905		Identification of Korotkoff sounds (*Nicolai Sergeivich Korotkoff*)
1926		Establishment of classic mercury sphygmomanometer
1930		Establishment of classic aneroid devices
1961		First ambulatory BP monitor (*AT Hinman*)
1976		First automated oscillometric BP device patent
1986		First guidelines for office BP measurement (*British Hypertension Society*)
1987		Establishment of validation standards for BP monitors (*US Association for the Advancement of Medical Instrumentation; British Hypertension Society*)
1995		First guidelines for ambulatory and home BP monitoring (*American Society of Hypertension*)
2005		Ambulatory and home BP monitoring included in algorithm for diagnosing hypertension (*Canadian Hypertension Education Program*)
2011		Ambulatory BP monitoring recommended for diagnosing hypertension (*UK National Institute of Health Excellence*)

**Table 2:** Chronological steps in the development of major antihypertensive drugs

1930s	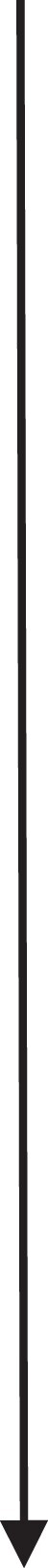	Veratrum alkaloids (*from the Lily plant family)*
1940s		Thiocyanates
		Ganglion blockers (*Tetraethylammonium*)
		Catecholamine depletors (*Reserpine—Rauwolfia*)
1950s		Vasodilators (*Hydralazine*)
		Peripheral sympathetic inhibitors (*Guanethidine*)
		Spironolactone
		Thiazide diuretics (*Chlorothiazide*)
1960s		Central adrenergic-2 agonists (*Methyldopa*, *Clonidine*)
		β-Blockers (*Propranolol*)
		Non-dihydropyridine calcium channel blockers (*Verapamil*)
		Thiazide-like diuretics (*Indapamide*)
1970s		Alpha1-adrenergic-blockers (*Prazosin*)
1980s		Angiotensin-converting enzyme inhibitors (*Captopril*)
		Dihydropyridine calcium channel blockers (*Nifedipine*)
1990s		Angiotensin II type 1 receptor blockers (*Losartan*)
2000s		Direct renin inhibitors (*Aliskiren*)

**Table 3:** Summary of landmark clinical trials that impacted hypertension treatment

Study	Design (medications)	Main conclusion	PMID
Hamilton *et al*. (1964)	Antihypertensive treatment in prevention of strokes and vascular complications	First controlled trial of antihypertensive therapies showing a significant reduction in strokes and other complications	14090850
VA-1st (1967)	Hydrochlorothiazide, reserpine, and hydralazine vs. placebo	Mortality and morbidity benefit, reduced progression to malignant HTN in patients with severe diastolic HTN (115–129 mmHg)	4862069
VA-2nd (1970)	Hydrochlorothiazide, reserpine, and hydralazine vs. placebo	Mortality and morbidity benefit, in moderately severe HTN (diastolic 90–115 mmHg)	4914579
HDFP (1979)	Stepped care vs. usual care; drugs: chlorthalidone, diuretics, reserpine, methyldopa, hydralazine, guanethidine	First study showing mortality and morbidity by aggressive, goal-directed BP management using incremental therapy in contrast to therapy with no target BP. This study set the ground rules for future management of HTN using incremental therapy—a new concept in managing chronic diseases	490882
MRC (1985)	Bendroflumethiazide or propranolol vs. placebo in mild diastolic HTN	Reduction in strokes and all CV events but not total mortality	2861880
EWHPE (1986)	Hydrochlorothiazide/triamterene vs. placebo	First major RCT in elderly population showing decrease in MI/cardiac deaths	3475430
SHEP (1991)	Chlorthalidone with a step-up to atenolol or reserpine	First RCT in isolated systolic HTN, which had been considered benign before	2046107
MRC (1992)	Elderly patients (65–74 years old) randomized to diuretic, β-blocker, or placebo	Hydrochlorothiazide and amiloride reduce the risk of stroke, coronary events, and all cardiovascular events in older hypertensive adults	1445513
TOMHS (1993)	Chlorthalidone vs. acebutolol vs. doxazosin vs. amlodipine vs. enalapril (all combined with nutritional/hygienic advice) vs. placebo/nutritional advice alone	Comparison of 5 classes and antihypertensive medications in addition to nutritional/hygienic advice	8336373
SYST-EUR (1997)	Nitrendipine with the possible addition of enalapril and hydrochlorothiazide or matching placebos in patients (>60 years old) with SBP 160–219 mmHg	First CCB RCT—in elderly with isolated systolic HTN, nitrendipine reduces the rate of cardiovascular complications	9297994
DASH (1997)	Three diet regimens including combination DASH diet	DASH diet reduced BP in both hypertensive and normotensive groups	9099655
HOT (1998)	Three DBP targets: ≤90; ≤85, or ≤80 mmHg; achieved by stepwise therapy with felodipine 5–10 mg/day; add ACE inhibitor or β-blocker then add thiazide diuretic	Large RCT; no significant difference in outcome between all three groups although all achieved DBP < 85 mmHg	9635947
UKPDS (1998)	BP control comparison with tight control (BP target < 150/85 mmHg), vs. less tight (< 180/105 mmHg) using captopril and atenolol	In T2D tight BP control achieved more macro- and micro-vascular morbidity and mortality benefits than tight blood glucose control	9732337
			9732338
Syst-China (2000)	Nitrendipine with the possible addition of captopril and/or hydrochlorothiazide in patients (>60 years old) with SBP 160–219 mmHg	Stepwise antihypertensive drug treatment improved prognosis, with particular benefit in patients with T2D	10647760
DASH (2001)	Low sodium DASH diet	Established sodium dietary levels in BP control	11136953
LIFE (2002)	Losartan vs. atenolol	Losartan confers benefits beyond reduction in BP and is better tolerated than atenolol	11937178
AASK (2002)	Compared different BP goals. Mean BP ≤92 mmHg vs. BP≤102– 107 mmHg).in African Americans using metoprolol/ramipril/amlodipine to slow down renal failure	Tighter BP control failed to reduce GFR decline although hypertensive nephrosclerosis was non-significantly slowed down	12435255
ALLHAT (2002)	Angiotensin-converting enzyme inhibitor (ACE-I; lisinopril), calcium channel blocker (amlodipine), and alpha-blocker (doxazosin) vs. thiazide-like diuretic (chlorthalidone)	Largest antihypertensive trial. Use of thiazide-like diuretics should be preferred at the start of therapy unless contraindicated	12479763
ANBP2 (2003)	Thiazide vs. ACE-I	Non-significantly different outcomes; Australia	12584366
VALUE (2004)	Valsartan vs. amlodipine in cardiac morbidity and mortality reduction in hypertensive patients at high cardiovascular risk	No difference in cardiac outcomes between treatments. Importance of prompt BP control in hypertensive patients at high cardiovascular risk	15207952
ASCOT (2005)	Atenolol/thiazide vs. amlodipine/perindopril	CCB/ACE-I combination gives much better outcomes than older regimen of β-blocker and hydrochlorothiazide	16154016
HYVET (2008)	(Indapamide±perindopril) vs. placebo in very elderly (≥80 years)	Mortality, CV events (including stroke) reductions; heart failure reduced by 64%	18378519
ACCOMPLISH (2008)	Comparison of ACE-I/CCB vs. ACE-I/diuretic	Benazepril + Amlodipine—19.6% relative risk reduction of composite CV death/CV events (including strokes)	19052124
ACCORD (2010)	Compare intensive BP lowering <120 mmHg to conventional BP < 140 mmHg in T2D	No significant benefit for intensive BP lowering in diabetics	20228401
SPRINT (2015)	Compare intensive BP lowering <120 mmHg to conventional BP < 140 mmHg in non-diabetics without prior strokes	Intensive lowering of systolic BP goal <120 mmHg in non-diabetic population report substantial reduction in major cardiovascular events with intensive BP lowering	26551272
STEP (2021)	Compare intensive BP lowering to 110–130 mmHg to a target of 130–150 mmHg in patients aged 60–80 years	In older patients, intensive lowering of systolic BP to 110–130 mmHg showed a lower incidence of cardiovascular events than standard the traditional target	34491661

PMID, Pubmed ID; HTN, hypertension; BP, blood pressure; CV, cardiovascular; RCT, randomized clinical trial; CCB, calcium channel blocking agent; DASH, dietary approaches to stop hypertension; DBP, diastolic blood pressure; T2D, type 2 diabetes mellitus; GFR, glomerular filtration rate; ACE-I, angiotensin-converting enzyme inhibitors. Trial full names listed in Pubmed papers.

**Table 4:** Summary of the development of hypertension guidelines

WHO/ISH	BHS/NICE	ESH	ACC/AHA	Canada	JSH
			NHLBI (JNC-1–7) 1977 **JNC-1–**1993 **JNC-5**		
				1984 Recommendations for mild hypertension 1985 Hypertension for the elderly 1988 Hypertension and diabetes 2000	
**WHO/ISH 1999** Definition of hypertension: ≥140/90 BP goal: 130/85 1st line medication: ARB, ACE-i, CCB, diuretics, α-blocker, β-blocker			1997 **JNC-6** BP target General: <140/90 Age >60 years: <140/90		**JSH2000** BP target of the old: <140–160/90
**WHO/ISH 2003** BP target Low, intermediate risk: SBP < 140 High risk: <130/80 Low-dose diuretics is recommended Cost-effectiveness	**2004-BHS IV** Focusing on ABPM and HBPM Medication: AB/CD rule	**ESH2003** BP target: General <140/90 Diabetes: <140/90 First-line medication: ARB, ACE-i, CCB, diuretics, β-blocker	2003 **JNC-7** Prehypertension (120–139/80–89 mmHg) BP target General: <140/90 Age >60 years: <140/90 Diuretics is the only first-line drug	Launch of **CHEP** Improvements in awareness and treatment rate Effective BP lowering at a population level	**JSH2004** Definition of hypertension using home BP and ABPM BP target of the old: <140/90
**2013 ISH/ASH** BP target General: <140/90 Age > 80 years: <150/90	**2011 BHS/NICE** Focusing on ABPM and HBPM BP target <80 years: <140/90 ≥80 years: <150/90 First-line dedication <55 years: ACE-I ≥55 years: CCB β-blocker is not recommended 2019 NICE guideline	**ESH2007** BP target, General <140/90 Diabetes: <130/80 First-line medication: ARB, ACE-i, CCB, diuretics, β-blocker	2014 **JNC-8** BP target, General: <140/90 Age≥60years: <150/90	2010 Hypertension Canada	**JSH2009** Comparison of home BP with office BP and ABPM first-line medication: ARB, ACE-I, CCB, diuretics, β-blocker Combination of diuretics and β-blocker is not recommended
		**ESH/ESC2013** BP target, General: <140/90 Age≥60years: <150/90 Diabetes: <140/85 First-line medication: ARB, ACE-i, CCB, diuretics, β-blocker	**2015 AHA/ACC/ASH** BP target: 140/90	2015 **CHEP recommendation** Preferential use of electronic upper arm device Diagnosis of hypertension based on out-of-office BP 2016	**JSH2014** Priority of home BP over office BP BP target, General: <140/90 *＞*75 years: <150/90 Diabetes: <130/80 First-line medication: ARB, ACE-I, CCB, diuretics
**ISH 2020 global hypertension practice guidelines** For application in both low-and high- resource settings	**Hypertension in adults: diagnosis and management**	**ESC/ESH2018** BP target General: <140/90 Age≥60 years: <150/90 Lower limit: DBP 170 First-line medication: RAS inhibitor (ARB or ACE-i) + CCB or diuretics Recommendation of SPC	**2017 ACC/AHA** Change in the definition of hypertension (≥130/80) BP target, General: <130/80 *＞*65 years: <SBP 130	**Hypertension Canada Guidelines**	**JSH2019** Categorization of BP value by home BP BP target, General: <130/80 *＞*75 years: <140/90 Diabetes: <130 < 80
**2021 WHO Guidelines for the Pharmacological treatment of Hypertension in Adults** Administration of treatment by non-physician professionals				**Hypertension Canada’s 2020 Comprehensive guidelines** AOBP > 135/85 included in office visit assessment of BP BP target: non-diabetic CKD, >50 years, elevated CV risk with SBP 130–180: <120 (AOBP)	

ABPM, ambulatory blood pressure monitoring; ACC, American College of Cardiology; ACE-I, angiotensin-converting enzyme inhibitor; AHA, American Heart Association; AOBP, automated office blood pressure; ARB, angiotensin II receptor blocker; ASH, American Society of Hypertension; BHS, British Hypertension Society; BP, blood pressure; CCB, calcium channel blocker; CHEP, Canada Hypertension Education Program; CKD, chronic kidney disease; CV, cardiovascular; ESC, European Society of Cardiology; DBP, diastolic blood pressure; ESH, European Society of Hypertension; HBPM, home blood pressure monitoring; ISH, International Society of Hypertension; JNC, Joint National Committee; JSH, Japanese Society of Hypertension; NHLBI National Heart, Lung, and Blood Institute; NICE, National Institute for Health and Clinical Excellence; RAS, renin–angiotensin system; SBP, systolic blood pressure; SPC, single-pill combination; WHO, World Health Organization

### 2.1 Blood pressure-lowering agents^22–24^

The era of antihypertensive drug development started in 1930 (*Table 2*), albeit with drugs that had unpredictable efficacy and frequent adverse effects. In the 1940s and 1950s, ganglion-blocking agents, hydralazine, and chlorothiazide were increasingly used and showed some evidence of the benefits of reducing BP. Only thiazide diuretics among these early antihypertensive drugs have survived as first-line agents until today. In the 1960s, central blockers of the sympathetic nervous system were developed, followed by β-blockers and later in the 1980s, calcium channel blockers (CCBs), the latter being one of the mainstays of current management. In 1977, angiotensin-converting enzyme inhibitors (ACEi) were synthesized and since then this drug class has played a major and increasing role in hypertension management and cardiovascular medicine. These drugs, together with the angiotensin receptor blockers (ARBs) which followed, remain the cornerstone of antihypertensive therapy for many patients. In the last 20 years, several new antihypertensive agents have been investigated for primary hypertension, including endothelin inhibitors, central renin–angiotensin system blockers, endothelial dysfunction modulators, and new aldosterone antagonists, and while none of them are recommended as first-line agents, spironolactone has been established as fourth-line therapy, optimal therapy for patients with resistant hypertension, and first-line therapy for primary aldosteronism.^25^

### 2.2 Non-pharmacological management of hypertension

Lifestyle improvement is a cornerstone of hypertension prevention, control and reducing the risk of CVD, and is recommended in all major guidelines.^25–29^ Dietary and lifestyle changes such as weight control, alcohol consumption reduction, smoking cessation, reduced dietary salt intake, and increased regular exercise not only improve cardiovascular health but reduce BP and improve hypertension control.^30–36^ Of non-pharmacological interventions, one of the most efficacious in lowering BP is the Dietary Approach to Stop Hypertension (DASH)^37–39^ underscoring the importance of a diet rich in fruits, vegetables, and low-fat dairy foods with reduced saturated and total fat, and reduced salt.^40^ Comprehensive lifestyle modification, when used as a combined intervention, provides added benefit.^40^

### 2.3 Landmark trials^41–45^

The results of early clinical trials in hypertension provided insights into both our understanding of the complications of high BP and optimal treatment. Hypertension pharmacotherapy is arguably the most studied evidence-base of any clinical intervention. Multiple seminal trials have shown that BP reduction is effective at reducing cardiovascular morbidity and mortality. Initially, diastolic BP was considered to be the primary target, as reflected by trial inclusion criteria, but soon, trials such as SHEP and SYST-EUR (*Table 3*) showed that treating isolated systolic hypertension is beneficial and in general, thereafter, more emphasis has been placed on systolic pressure as inclusion criteria for trials. As trial inclusion criteria changed, the definition of hypertension followed the evidence from being initially based only on diastolic BP to include systolic criteria and also changed from ≥160/90 to 140/90 mmHg.^46^ Other key questions followed regarding optimal treatment targets. This was addressed by a series of trials most recently the SPRINT trial (*Table 3*), which have informed gradually lower targets. A further pivotal question was—what is the best way of achieving these targets? The stepwise incremental therapy approach (‘Stepped care’) was introduced in the Hypertension Detection and Follow-Up (HDFP) trial in 1979 (*Table 3*) and was tested leading to huge improvements in clinical outcomes and survival. Finally, the question as to which drugs were the most effective in terms of preventing CV outcomes was assessed in a series of trials comparing older and newer medications and their combinations (e.g. ALLHAT, ASCOT-BPLA, LIFE, ANBP2, ACCOMPLISH, VALUE, etc.) (*Table 3*). Recently, the situation has changed, with all major hypertension guidelines now recommending single-pill combination (SPC) therapy as first-line treatment—a major update in the therapeutic approach to hypertension.

### 2.4 Guidelines

The definition or diagnostic threshold of hypertension changed from an office BP of ≥160/90 to ≥140/90 mmHg in 1993,^46^ based on benefits of treating lower BP levels in outcome trials (*Table 4*). In part reflecting the results of SPRINT (*Table 3*), the diagnosis of hypertension was lowered to 130/80 mmHg in the American College of Cardiology/American Heart Association (ACC/AHA) guideline in 2017.^27^ However, other guidelines thereafter^29,47^ have maintained the previous diagnostic threshold of ≥140/90 mmHg.

As thresholds were lowered to ≥140/90 mmHg, BP targets also fell to <140/90 mmHg. However, as further BP lowering achieved a greater reduction in cardiovascular events in hypertensive patients with comorbidities and complications,^45^ the 2007 European Society of Hypertension/European Society of Cardiology (ESH-ESC) hypertension guideline^48^ lowered the BP goal to <130/80 mmHg and other guidelines^49,50^ also set the goal as <130/80 mmHg for hypertension associated with diabetes. Moreover, the apparently negative result of ACCORD-BP (*Table 3*), resulted in more conservative guideline recommendations for BP targets in patients with diabetes of <140/85 mmHg^51^ and 140/90 mmHg,^52–54^ whilst Japanese guidelines remained unchanged at <130/80 mmHg^55^ and subsequent guidelines^29,56^ have lowered BP targets to <130/80 mmHg for most patients younger than 65 years.

Different BP targets have been set for different age groups and these targets have also changed over time. In 1993, the Joint National Committee on Prevention, Detection, Evaluation and Treatment of High Blood Pressure (JNC-5)^46^ took a negative stance on BP lowering for those aged ≥65 years, but in 2003, JNC-7^49^ set the same target of <140/90 mmHg for those aged at and above or below 65 years based on the SHEP and Syst-Eur trials (*Table 3*). Presumably based on the targets and treatment benefits shown in the HYVET trial among those aged ≥80 years (*Table 3*), the target BP was then raised to 150/90 mmHg in later guidelines^52,53^ only to be lowered again^27,29^ following the results of SPRINT (*Table 3*).

The use of out-of-office BP monitoring for hypertension diagnosis and treatment decisions is increasingly recognized worldwide and is now strongly recommended by most hypertension guidelines.^25,27,29,56^ Guidelines first mentioned out-of-office BP levels in 1995,^57^ but only in 2005^58^ did a guideline include out-of-office thresholds for diagnosis. In 2011, ambulatory BP monitoring (ABPM) was recommended as necessary to diagnose hypertension,^59^ a controversial proposal but one which has increasingly gained traction thereafter, with differing proponents of home BP monitoring (HBPM)^55^ and ABPM. Clearly, resources impact the utility and suitability of the routine use of ABPM and HBPM.^47^ More recently, digital and e-health strategies are being recommended to track and inform lifestyles and wellbeing together with BP levels of patients with hypertension.^60^

Recommendations for first-line medications have varied, sometimes according to age and ethnicity,^61^ but prior to 2003 CCBs, ACE-Is, ARBs, diuretics, α1-blockers, and β-blockers were recommended.^62–64^ Subsequently, α1-blockers and β-blockers were dropped from first-line antihypertensive treatment due to less effective cardiovascular protection.^29,59^ However, WHO/ISH^65^ and JNC-7^49^ only recommended diuretics as first-line medication based on results of the ALLHAT study (*Table 3*). More recently, several guidelines^29,47,66,67^ have recommended that antihypertensive medication should start with SPCs to ensure more rapid, effective BP-lowering, although the combinations recommended have varied across these contemporary guidelines.

The importance of inclusivity and interprofessional collaboration to generate shared decision-making is highlighted in recent Canadian guidelines,^68^ whilst the latest WHO guidelines^66^ emphasize the use of a task-sharing approach for hypertension management in low-resource settings.

There is also increasing emphasis on the importance of monitoring and promoting adherence to antihypertensive treatment.^29,47^

### 2.5 Treatment and control

Reports of BP surveys in the US spawned the term ‘rule of halves’ in a 1972 publication^69^ whereby:

- half of those with hypertension (defined as ≥160/95 mmHg at the time) were aware of it,
- half of those aware of having hypertension were treated, and half of those on treatment for hypertension were controlled. Hence, ∼12.5% of all ‘hypertensives’ were controlled to <160/95 mmHg.

Serial data from representative samples of the US adult population between 1971 and 1991^70^ showed significant improvements in these parameters (still based on a 160/95 mmHg threshold) such that among all hypertensives, awareness rose from 51 to 84%, treatment from 36 to 73%, and control from 16 to 55%.

A series of five surveys of representative samples of adults in England between 1994 and 2011 showed awareness rose from 46 to 71% treatment from 32 to 58% and control from 11 to 37% based on the more aggressive diagnostic threshold and target of 140/90 mmHg.^71^

Again, using the 140/90 mmHg cut-point, global data since 2000 show variable results depending on regional income status but between 2003 and 2009, global awareness remained <50% and although treatment rates were much higher at 87%, only 13% of those aware were classified as controlled.^72^

Other global reviews of more contemporary data (2008–16) in HICs show awareness rates of ∼71%, treatment rates of almost all those aware (86%) but control rates of only ∼37% of all hypertensives.^73^

The improvements in awareness, treatment, and control rates in several HICs have reportedly plateaued in recent years. In LMICs, contemporary (2005–16) levels of awareness are worse than in HICs at 39%, of whom 76% were treated, and overall, only ∼10% of all hypertensives were controlled.^74^

**Figure 3:**
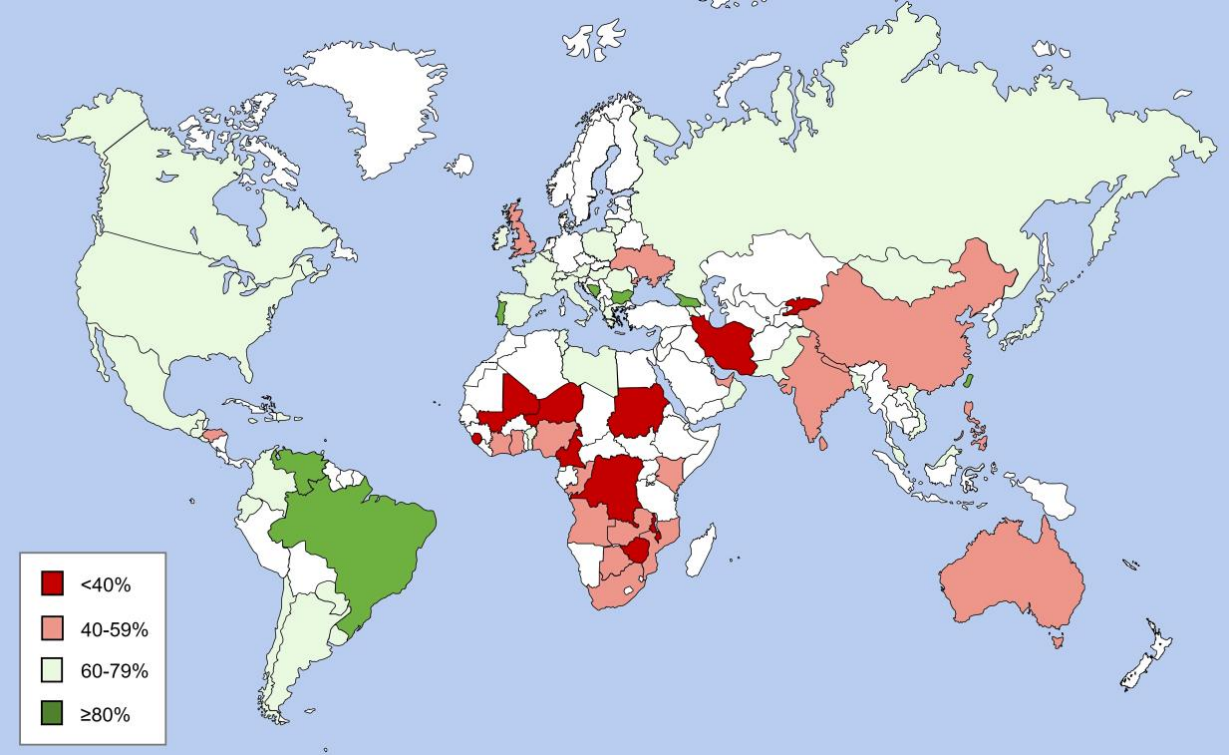
Awareness rates of hypertension in 83 countries participating in opportunistic screening as part of the May Measurement Month campaign. Combined data for 2018 and 2019 in >2.7 million, with screenees per country ranging from 500 to 701 566. Only countries with at least 500 screenees are included.

**Figure 4:**
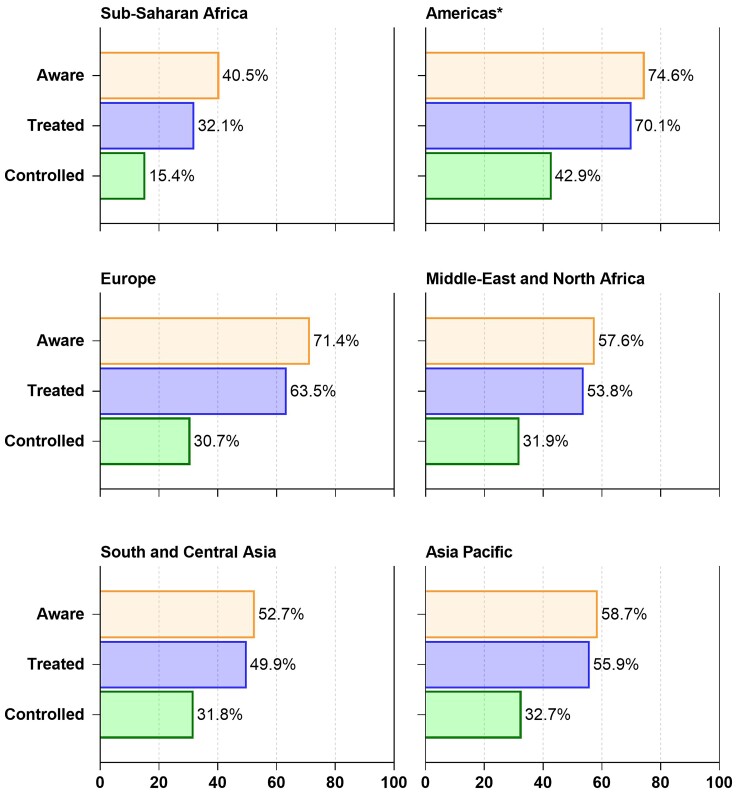
Awareness, treatment, and control rates of MMM screenees defined as hypertensive (2018 and 2019). Percentage of screenees who were hypertensive: *Sub-Saharan Africa:* 26.6% of total (*N* = 370 395) screened. *Americas:* 40.8% of total (*N* = 449 117) screened *excluding the USA and Canada. *Europe:* 43.1% of total (*N* = 186 159) screened. *Middle-East and North Africa:* 28.3% of total (*N* = 139 908) screened. *South and Central Asia:* 31.3% of total (*N* = 864 394) screened. *Asia Pacific:* 34.1% of total (*N* = 1 001 001) screened.

The May Measurement Month (MMM) global BP awareness campaign reports the most contemporary data (2017–19) of awareness, treatment, and control rates across over 100 countries but used opportunistic and hence non-representative sampling. (See *Figures 3* and *4*.)

## 3. The impact of raised BP across the globe

The first step towards improving the hypertension cascade is to increase awareness. Although seemingly logical, this recommendation was pivotal in the Lancet Commission on hypertension report, namely ‘Every adult should know their blood pressure’.^13^ In keeping with this recommendation, the ISH launched the annual MMM campaign globally to increase awareness. This was an expansion of World Hypertension Day held annually on 17 May as initiated by the World Hypertension League. Based on over 2.7 million people screened in 2018 and 2019, it is clear that the vast majority of people with raised BP are unaware (*Figure 3*), with evident inequities apparent between many HIC and LMICs—albeit based on opportunistic screening (Methods in Supplementary material online).

In the subsequent section, brief summaries are given for each global region, with insights on the epidemiology, unique challenges, and opportunities to improve BP control.

### 3.1 Sub-Saharan Africa

Countries: Angola, Benin, Botswana, Burkina Faso, Burundi, Cabo Verde, Cameroon, Central African Republic, Chad, Comoros, Congo, Cote d’Ivoire, Democratic Republic of Congo, Equatorial Guinea, Eritrea, Eswatini, Ethiopia, Gabon, Gambia, Ghana, Guinea, Guinea-Bissau, Kenya, Lesotho, Liberia, Madagascar, Malawi, Mali, Mauritania, Mauritius, Mozambique, Namibia, Niger, Nigeria, Rwanda, Sao Tome and Principe, Senegal, Seychelles, Sierra Leone, Somalia, South Africa, South Sudan, Sudan, Togo, Uganda, United Republic of Tanzania, Zambia, Zimbabwe.

#### 3.1.1 Epidemiology

During the last few decades, SSA has shown a constant increase in the prevalence of hypertension, without any significant improvement in the levels of awareness, treatment, and control. The MMM campaigns of 2018 and 2019 demonstrated that, when compared with other regions in the world, SSA had lower levels of awareness, treatment, and control of hypertension: 40.5%, 32.1%, and 15.4% respectively (*Figure 4*). Unfortunately, MMM may not represent the underlying population because sampling was by convenience. However, in a recently published analysis of more than 104 million participants from population-representative studies, SSA shared with Oceania and South Asia the lowest levels of awareness, treatment, and control of hypertension in the world, much lower than the ones described in the MMM analyses.^75^

#### 3.1.2 Challenges

- A major challenge in SSA is that, as one of the most under-developed regions in the world, it faces considerable poverty. Geographical distances and political instability can significantly limit access to healthcare and emergency medical services.^76^ This is coupled with inadequate healthcare systems with limited guidelines.
- Due to poverty but also due to cultural beliefs, many patients first see a traditional healer, which is much more accessible than a medical doctor. It has also been well described that traditional herbal medicines without any evidence for effectiveness are used either alone or together with allopathic medicines to treat hypertension.^77^
- Unfortunately, there is a dearth of information available for the region due to administrative restrictions and limitations to research.^78^ The lack of political will to provide access to healthcare, corruption, and limited healthcare system transparency restricts the ability to manage non-communicable diseases (NCDs). Furthermore, political leaders often deny NCD-associated health problems.
- Lack of funding means that healthcare professionals may lack the skills, training, and resources to manage hypertension. Limited healthcare system funding also means that BP-lowering medications (when available) are often self-funded but extreme poverty makes hypertension control unreachable for the majority.
- Education and literacy levels are low throughout SSA, raising concerns about population awareness of NCDs; a high degree of poor adherence to medication and suspicion towards Westernized healthcare compound these problems.^79^
- Another challenge on the continent is hypertension-related health disparities. Reasons for health disparity in Africa include socioeconomic status (SES) and educational background, location, religion, and colonial relationships. Depending on the dimensions under consideration, the disparities cut across countries, different regions of the continent, or even within countries. The situation within the continent is heterogeneous, with more developed countries like Mauritius, Seychelles, South Africa, and Namibia showing a level of BP control that is sometimes more than double that of poorer countries.^4^ Race and ethnicity are considered major contributors to health disparities, particularly in higher-income countries. In small populations, such as rural Pygmies that continue to follow hunter–gatherer lifestyles very low levels of urinary sodium excretion and very low BP levels are still reported.^80^ Cardiovascular risk in Africa appears to be greater due to a loss of these traditional lifestyles with a rapid transition towards western lifestyles, and a combination with genetic risk, leading to early hypertension-mediated organ damage seen during urbanization.
- In HIC, higher SES is associated with a lower prevalence of hypertension.^81^ In SSA, the association between hypertension and SES is less clearly defined with differences noted based on sex, including a lower prevalence in women who are consistently better treated and controlled than men.^82^ There are disparities in risk factors associated with hypertension, such as salt consumption, obesity, alcohol intake, and cigarette smoking. A wide range in alcohol consumption exists across different countries in Africa.^83^ Within these countries, cultural and religious beliefs also affect reported alcohol consumption, including systematic under-reporting of and in enacting the legislature against alcohol.
- The prevalence of obesity has been on the increase in the entire sub-region. However, the rate of increase varies widely across different regions. Between 1990 and 2015, the prevalence of those overweight tripled from 6.4 to 21% in the Southern African region, while in Eastern Africa, the prevalence increased marginally from 4.5 to 5%. In-country prevalence of obesity varies according to a person’s SES with those in higher-income classes more likely to be obese.^84–87^
- Access to antihypertensive medication and hypertension-related health care services vary widely within and across countries.^88^ The national essential medicine list in various countries is not regularly updated in line with WHO Essential Medicines List (EML) and as such guideline-recommended therapies may not be adhered to, a practice necessary to ensure BP control. Generally, Africa has low availability of hypertension services, but this is particularly evident in rural areas.
- Due to the well-documented shortage of physicians in SSA, the task-sharing strategies, whereby non-physician health workers adopt roles that include the diagnosis and prescription of antihypertensive medications, have been found to be effective in controlling hypertension. These policies are yet to be adopted throughout SSA.
- Disparities in antenatal care may widen the gap in the burden of hypertension between the poor and the rich in later life. Huge differences exist in the burden of pre-eclampsia, malaria in pregnancy, and low birth weight across different regions of Africa. Earlier reports have indicated that individuals who developed in an unfavourable intrauterine milieu have a greater predisposition to early-onset hypertension.^13^

#### 3.1.3 Opportunities to improve BP control

African populations are particularly sensitive to salt intake.^89^ Although several global reports have noticed that salt consumption in Africa is low, the data are overall weak and there is a need to strengthen data on salt intake. More recent data show that in some cases, the consumption of salt is double the level recommended by the WHO with a large amount of discretionary added salt.^90^ With the recent evidence of the beneficial effects of salt substitutes,^91^ this could be a cost-effective way for primary prevention of hypertension.

In terms of primary prevention, infant and maternal health programs have strong external support. The correct diagnosis of hypertension during pregnancy and the prevention of pre-eclampsia, which are extremely common in SSA, could also reduce the long-term consequences of chronic hypertension, stroke, heart failure, acute myocardial infarction, and chronic kidney disease (CKD).^92^

Hypertension may be defined as a health priority, but, in practice, the limited national funding is preferentially allocated to infectious disease and maternal/child health programmes. The result is that NCD care is dependent on donations and competes for funding with infectious disease and HIV programmes. The integration of NCD clinics with HIV care has already proven to be effective. This partnership, if well used, would increase the levels of awareness, treatment, and control, mostly in the eastern and south region of Africa where HIV is still extremely prevalent.

The first step in addressing the challenges associated with the limited hypertension healthcare in Africa is an acknowledgement by funders that healthcare is a basic right and action plans need to be put in place to provide access to healthcare. This includes a strong emphasis on strengthening primary healthcare (as a global WHO priority), including staffing, training, availability, and affordability of good quality generic medications, mobile clinical services, nurse-led services, and engaging with traditional healthcare providers.

Specific opportunities include:

- Decrease salt consumption with national policies to decrease the amount of sodium in food and promote the use of potassium salt substitutes.
- Integrate hypertension control into well-established HIV or maternal and child health programmes that are financed and functioning well.
- Team-based care, not only with nurses but by using innovative approaches involving community health workers, lay people, and even traditional healers.
- Roll-out and scale-up of the WHO HEARTs package as a model to strengthen primary care using a public health approach.

### 3.2 The Americas

Countries: Antigua and Barbuda, Argentina, Bahamas, Barbados, Belize, Bolivia, Brazil, Canada, Chile, Colombia, Costa Rica, Cuba, Dominica, Dominican Republic, Ecuador, El Salvador, Grenada, Guatemala, Guyana, Haiti, Honduras, Jamaica, Mexico, Nicaragua, Panama, Paraguay, Peru, Saint Kitts and Nevis, Saint Lucia, Saint Vincent and the Grenadines, Suriname, Trinidad and Tobago, USA, Uruguay, Venezuela

#### 3.2.1 Epidemiology

The Americas population comprises over 1 billion persons among 36 diverse countries over a land area of 42 million km^2^. Hypertension surveillance surveys use varying measurement techniques, differing cut-point definitions of hypertension and bear varying degrees of completeness across countries.^4,93–97^ Using a similar ≥140/90 mmHg cut-point for hypertension, prevalence ranges from a high of 43% in Latin America^93^ to 29% in the USA^4,94^ and 23% in Canada^95^ with men generally having a higher prevalence than women.^93,94^ Special mention should be made of the Caribbean countries where hypertension prevalence has continued to increase, with figures exceeding 45% in some countries.^4,18^ Awareness of hypertension is generally high throughout the Americas (83% USA, 77% Canada, and 63% in Latin America) (*Figures 3* and *4*).^4,93–97^ Among those aware of their diagnosis, 93% in Canada, 73% in the USA, and 49% in Latin America were treated. Overall, among those with hypertension, control is suboptimal (58% in Canada, 51% in the USA, and 21% in Latin America) and with the pandemic, control may have fallen further.^98^

#### 3.2.2 Challenges

The main challenges to achieving hypertension control include a poor diet, increased sedentariness, rising obesity, and limited access to health services and low-cost medications.

- Approximately 10% of the US population is without affordable healthcare insurance^99^ and the uninsured/underinsured are more likely to have poor BP control.^100^
- Another principal cause for suboptimal hypertension control is medication non-adherence and therapeutic inertia. In Canada, despite a universal healthcare system and access to low-cost medications, almost 50% of patients with hypertension are non-adherent to medications.^101^ In Latin America, treatment inertia remains a problem, where there is suboptimal intensification of therapy with 65% of treated patients only prescribed one antihypertensive agent.^93^
- Disparities in BP control in the Americas also pose a challenge and arise from multiple sources. These include differences in healthcare systems, SES inequalities within countries, limited healthcare access in rural areas, and disparities in immigrant and visible minority populations compared with White populations. Countries within the Americas have differing models of healthcare with Canada having universal healthcare, and the highest hypertension control rates. The US healthcare system is largely privatized whereas Latin American countries provide both systems with ∼80% receiving publicly funded care.^102^ In a meta-analysis of differing payer systems, reduced co-payments for healthcare, including medications, were associated with improved outcomes of hypertension.^103^ However, similar control can be achieved in HICs or middle-income countries, from the private sector or fully public initiatives, indicating that BP inequalities can be overcome with specific strategies.^104,105^ SES inequalities within countries are also associated with differences in hypertension control. Within the USA, hypertension prevalence was lowest among college graduates (39%) vs. those having a high school education or less (47%) and those with more than high school or some college (51%).^94^ Notably ∼30% of adults with hypertension without health insurance were unaware of their hypertension compared with 14% of those insured. As healthcare visits increased, the percentage of adults with hypertension who were unaware of their status decreased.^100^
- Within countries, there is considerable variation in hypertension incidence, control and outcomes between rural and urban dwellers, immigrant vs. non-immigrant groups, and ethnic groups. The incidence of hypertension increased in rural populations, especially the rural southern USA^106^ with 40% of adults in rural areas reported to have hypertension, compared with 29% in urban areas.^107^ This disparity is thought to be due to less access to healthcare and a lack of transportation in rural areas. In Canada, non-Hispanic black, and South Asians have a higher prevalence of hypertension and a younger age of onset compared with other groups.^108^ In the USA, hypertension prevalence remains higher among non-Hispanic Black (57%) than non-Hispanic White (44%) adults (applying the threshold of 130/80 mmHg).^94^ Non-Hispanic Black and Hispanic patients also had worse hypertension control than non-Hispanic White patients.^109,110^ Hypertension accounts for 50% of the racial differences in mortality between non-Hispanic Black and White populations in the USA.^109^ However, some ethnic groups possess a low risk of hypertension. The Kuna Indians of Panama exhibit an exceedingly low prevalence of hypertension (2%) thought to be secondary to a diet high in fruit, fish, low salt, and cocoa beverages compared with other Panamanians and Kuna Indians who transitioned to urban areas.^14^ Disparities vary by the ethnic group but are likely related to social determinants of health, access to care, salt sensitivity, and increased adiposity.^111^

#### 3.2.3 Opportunities to improve BP control

Initiatives are needed to improve hypertension control that address BP disparities in underserved and marginalized populations using culturally appropriate approaches and improving practice level quality. Here, we outline four initiatives that could be scaled to a population level to achieve large impacts on BP control.

- Culturally tailored health promotion in non-traditional settings: Barbershops were used for health promotion to encourage African-American men to visit prescribing community pharmacists working in collaboration with physicians compared with encouraging patrons to visit their physician. Leveraging community pharmacists through trusted community lay people in a culturally specific environment was associated with superior BP control.^112^
- Improving the quality of care in hypertension: The Kaiser Permanente model with simple algorithms, using SPCs, registries to monitor hypertension control rates at a practice level, feedback tools, and multi-disciplinary approach was associated with dramatic improvements in hypertension control compared with historical usual care.^113^ This informed the WHO HEARTS Technical package for wide-scale implementation.
- Optimizing virtual hypertension care: Given the widespread adoption of virtual care, optimization of virtual care delivery and adoption are urgently needed including improving digital literacy, especially in marginalized communities, increasing equitable access to broadband internet, home telemonitoring, and reimbursement models for care providers. Implementation of home telemonitoring with automatic tele-transmission of BP measurements to their healthcare provider teams was associated with improved medication adherence and BP control.^114^
- Development of national, low-cost antihypertensive medication programmes, which is feasible, given the relatively low cost of currently recommended medications.

### 3.3 Europe

Countries: Albania, Andorra, Armenia, Austria, Azerbaijan, Belarus, Belgium, Bosnia and Herzegovina, Bulgaria, Croatia, Cyprus, Czech Republic, Denmark, Estonia, Finland, France, Georgia, Germany, Greece, Hungary, Iceland, Ireland, Israel, Italy, Latvia, Lithuania, Luxembourg, Malta, Monaco, Montenegro, Netherlands, North Macedonia, Norway, Poland, Portugal, Republic of Moldova, Romania, Russian Federation, San Merino, Serbia, Slovakia, Slovenia, Spain, Sweden, Switzerland, Turkey, Ukraine, UK

#### 3.3.1 Epidemiology

**Figure 5:**
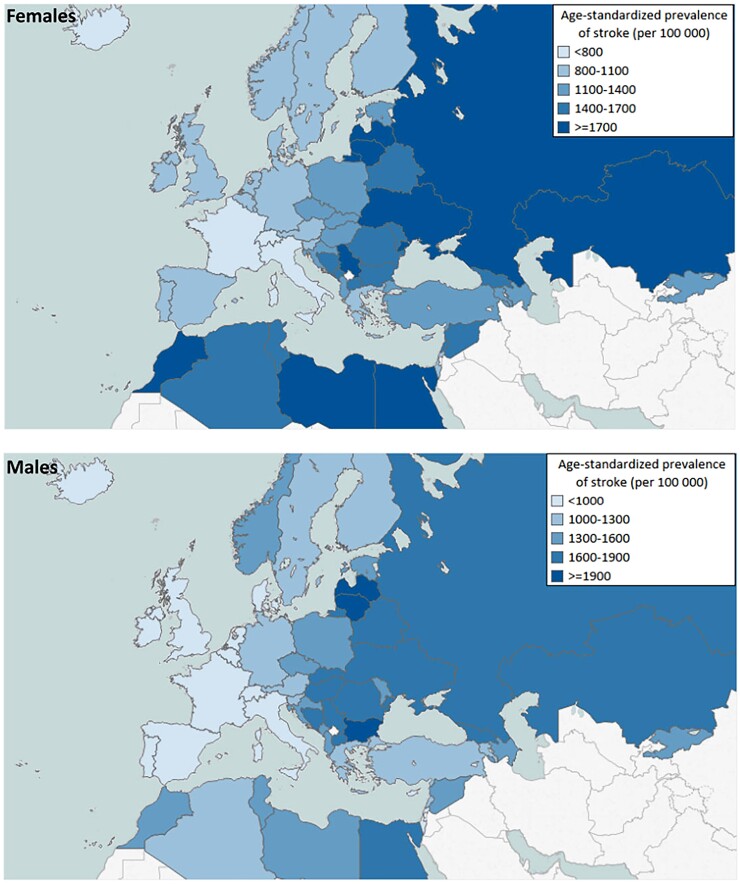
The West–East divide in Europe for stroke risk.^115^ (Republished with permission from Oxford University Press on behalf of the European Society of Cardiology. *Data source:* Institute for Health Metric Evaluation. All rights reserved.)

The prevalence of hypertension in Europe is strongly influenced by its ageing population and increased survival following cardiovascular events. Despite the documented improving trends for better BP control over the last 30–40 years, hypertension prevalence, awareness, and control in ageing European populations remains a considerable public health problem as recently documented again by the MMM screening campaign (*Figure 4*). A former North–South gradient of cardiovascular risk has been replaced by a West–East gradient during more recent decades,^115^ as exemplified by stroke (*Figure 5*).

This is mirrored by increasing prevalence rates of hypertension, stroke morbidity, and mortality in Eastern Europe and Russia.^116^ In most Western countries, there is a trend towards better control of BP in pharmacologically treated patients.^117–120^ This could represent more effective treatment, increased adherence, and better-educated patients seen in over the last 30–40 years. Although a further improvement of control rates is needed, the age-adjusted mean SBP is declining in Western Europe since the 1980s.^121^ Moreover, the age-adjusted prevalence rate of hypertension has not increased over recent decades.^4^ However, in Europe’s ageing population, the absolute number of patients with known hypertension has substantially increased, thereby imposing a heavy burden on healthcare in most countries.

In addition to the high-risk ageing population, other emerging risk groups are survivors of extreme preterm birth and congenital heart defects. Thanks to improved medical care in HICs, these individuals are now reaching adult age and will represent a novel group with strongly increased cardiovascular risk in the future.^122^

The West–East gradient in hypertension prevalence exists in both the female and male populations. A recent WHO-funded pooled analysis of data on BP levels and treatment in individuals aged 30–79 years derived from 1200 population-representative studies and covering the period from 1990 to 2019 revealed that the prevalence of hypertension in Eastern and some central European countries was 20–30% higher than in high-income Western European countries.^73^ While hypertension prevalence hardly differed between West and Central/East Europe and were generally high compared to the rest of the world (around 75% in all areas of Europe compared to only 59% worldwide), control rates differed significantly in these populations and were only 25% in women / 17% for men in central and Eastern Europe versus 45% in women / 37% for men in high-income Western countries (world wide control rates: women 23%, men 18%). Over the past three decades, despite observing an increase in the absolute number of hypertensive Europeans due to ageing and population growth, control rates have continuously improved for both sexes.^4,73^

#### 3.3.2 Challenges

- The high prevalence of untreated or poorly controlled BP in migrant populations has been studied in immigrants of Ghanaian,^123^ South-East Asian, or the Middle-East^124^ descent. Social problems, unemployment, and suboptimal standards of care are also important factors that challenge the care of hypertensive patients.
- The change of political regime in Eastern Europe has led to challenges in the health reform of these countries, which has greatly affected the control of BP.
- Another socially patterned problem is high alcohol consumption in many parts of Central and Eastern Europe, well known to associate with raised BP.
- Obesity, another determinant of hypertension, is also socially patterned and often combined with high alcohol intake (extra calories) in the same parts of Europe. Indeed, it appears that the burden of obesity increased in Central and Eastern Europe with the accession to the European Union.^125^

These social determinants of health contribute to the suboptimal BP control rates in most European populations despite health cost coverage and the wide availability of low-cost antihypertensive drugs.

- Ongoing political tensions and war in the Ukraine are anticipated to substantially impact local health systems and BP control in the region. The impact of war on cardiovascular health and hypertension care is widespread, with a direct relationship between combat wounds, war-related posttraumatic stress disorder, and increased risk of hypertension.^126^ However, the immediate and long-term effects of war on hypertension are apparent not only in service men and women but also has a direct impact on all affected people’s lives, safety, and freedom. The recent Russian invasion into an independent European state, Ukraine, is likely to translate into profound consequences on education, research, free movement, access to resources, breaking chains of medical supplies, and diagnostics affecting millions of lives. Due to the impact of this crisis, the ISH has published a message of solidarity with the Ukrainian people.^127^
- Salt intake is high in many European countries due to culinary traditions and easy access to processed food. This has influenced both prevalence of hypertension and the risk of stroke and other CVDs. In 2013, the WHO reviewed salt reduction activities across the European region, which prompted several countries, such as Portugal, to initiate health campaigns to reduce salt consumption and adopt legislative measures to reduce salt content in processed food,^128^ of special benefit to salt-sensitive people. While it is too early to assess the effectiveness of the measures in Portugal, the Finnish North Karelia Project, which started in the 1970s, was shown to be very effective. This programme, which combined community-based interventions, national-level policy changes, and legislation on lifestyle measures (e.g. salt and saturated fat intake reduction, smoking cessation), achieved marked reductions in population salt consumption, BP, and cardiovascular mortality in 35- to 64-year-old men and women by 82 and 84%, respectively.^129^

#### 3.3.3 Opportunities to improve BP control

In some European countries (e.g. in Poland), extensive nationwide screening involving BP is current practice.^130^ This has contributed to an improvement in BP control. Other initiatives aimed at establishing national registers for certain groups of patients, for example, with diabetes mellitus, while also following up on BP control and antihypertensive treatments. One such example is the National Diabetes Register from Sweden since 1996, including BP data, both from hospitals and from primary health care.^131^

The most important action to control BP in Europe is to have a well-structured and financed healthcare network around the hypertension patient involving mainly the primary healthcare physician, but also the hypertension specialist and pharmacist, among other caregivers of the patient for raising awareness, screening, diagnosis, treatment, and follow-up.^132^ An increased use of ABPM and home BP recordings can also contribute to better results,^133^ especially when integrated into digital health.^132^

Evidence-based recommendations for diagnosis and treatment of hypertension and risk stratification in women are lacking as a direct consequence of too few enrolled women. For Europe, this is exemplified by the HOPE study, which had 73% European participants, but only enrolled around 26% women.^134^

Prevention of hypertension and its complications in Europe should focus on improving social conditions and the working- and living environment (including programmes to prevent alcoholism) in underserved populations. It is also important to apply a life-course approach starting with preventive maternal and child healthcare. Adverse factors in the early life of premature and growth-retarded newborn babies may lead to increased risk of hypertension in adult life. Many health benefits will be obtained by reducing global cardiovascular risk throughout the entire lifespan, as also emphasized by the Lancet Commission on Hypertension.^13,135^

The geographical divide in cardiovascular risk in Europe, with higher incidence rates in Eastern Europe and Russia, calls for effective programmes involving recommendations for screening and treatment of hypertension. Finally, in ageing populations of Europe, many patients survive cardiovascular events and need effective treatment of remaining risk, including appropriate risk factor control. This also applies to the many comorbidities of elderly patients with hypertension, such as diabetes mellitus, CKD, and cognitive decline. The recommendation here is to have a multifactorial approach to reduce the overall risk based on guidelines. A summary of opportunities to improve BP control include:

- Support screening, diagnosis, treatment, and follow-up of hypertension throughout the healthcare network (involving primary care, pharmacists, nurses, and paramedics), especially in the ageing populations of Europe. In countries with high awareness, patients may be further empowered by mobile application supporting BP monitoring, adherence, and lifestyle advice.
- Tailor screening programmes for hypertension to overcome health disparities in age, gender, social background, ethnicity, and access to health care—also found in HICs of Europe.
- Provide simplified treatment strategies and effective risk factor control, where BP control is part of a general preventive strategy.
- Support hypertension programmes in Central and Eastern Europe to reduce the burden of hypertension complications and excess cardiovascular risk that contributes to the West–East divide in cardiovascular health of Europeans.
- Implement national and regional registers to follow trends in hypertension prevalence, awareness and control, both in the population and in specific groups of patients, i.e. established CVD, diabetes, or CKD.

### 3.4 Middle-East and North Africa

Countries: Algeria, Bahrain, Djibouti, Egypt, Iran, Iraq, Jordan, Kuwait, Lebanon, Libya, Morocco, Oman, Qatar, Saudi Arabia, Syria, Syrian Arab Republic, Tunisia, United Arab Emirates, Yemen

#### 3.4.1 Epidemiology

Hypertension is currently affecting ∼30% of the adult population in the Middle-East and North Africa (MENA) with wide variation between countries.^4,136^ This prevalence rate was also broadly reflected by the population of ∼140 000 participating in opportunistic screening as part of MMM (*Figure 4*), where only 31.9% of people with hypertension were controlled.

Within MENA, raised BP is causing an overburden on the medical system at governmental and individual levels as reported by the WHO.^137^ The early identification of hypertension requiring medical screening is more challenging in the region, as is estimating the representative incidence and prevalence rates for hypertension.

#### 3.4.2 Challenges

- The majority of the countries in the MENA region are classified as LMICs according to the World Bank definitions. In stable HICs, the burden of diagnosis and benefit of treatment of raised BP to the target levels required was clearly shown in early studies. The situation is very different in LMICs with limited health budgets and health manpower.^6^ Getting the population in such communities to accept preventive medicine and seeking health screening are challenging and need robust efforts to change beliefs and behaviours. At the same time, it remains challenging to provide accessible and affordable health services.^137–141^
- Wars and political instability have added an extra burden and led to deviating the attention towards the basics of living. Safety, food, and shelter became the main goals for a significant percentage of the population. Addressing screening and disease prevention became a commodity that has no place in the day-to-day living with the struggle for survival.^4,7,136,141^ Over the past decades, most of the regional countries were involved, directly or indirectly, in conflicts which in some cases were military confrontations. This generated a wave of immigration and refugees that impaired further the provision of proper health services in that population sector.^142^ This is true even when families had moved to HIC. These resettlements increased the health burden around the area of conflict, where the baseline health expenditure and budgets, in most cases, are already stretched.^143^
- When focusing on current practices to treat hypertension, these are highly ineffective and in the long term, will only increase the costs required to manage related organ damage and morbidities. The current measures and strategies are largely ineffective in many HIC, which illustrates that large-scale change in the detection and management of hypertension in HIC and LMICs are required.^4^ For that reason, urgent action plans are needed to expand allocated health budgets to be able to meet the increasing demands of managing raised BP and its complications. This is true for all countries within all economic levels.^136,139^
- Another critical aspect relevant to the MENA region is the limited amount of research being conducted in general. There are major barriers such as modest funds allocated to research, as well as having urgent priorities for both the government and individuals related to conflicts.^136^ When systematically reviewing the literature, there are only limited data generated usually from HICs in the region. Hence, a large percentage of these numbers are not representative of the entire region. This is also highly relevant for regional guidelines that are often derived from global recommendations designed for an entirely different population.^4,7^

#### 3.4.3 Opportunities to improve BP control

With all these challenges mentioned, how can the MENA region be assisted in the fight against hypertension and its consequences?

- Local adoption of guidelines to define and classify hypertension considering the regional socioeconomic status and accompanying challenges—as described in the 2020 ISH guidelines.^25^ It should be understood how challenging it is to manage hypertension in an LIC as opposed to a stable HIC, where healthcare staff have to deal with patients from varying populations, standards of living, and genetic variation, with minimal resources.
- Research data are limited from participants and countries in the MENA region. Greater efforts should be spent on creating and maintaining accurate and timely epidemiological data on prevalence, treatment, and control rates. This includes raising funds from regional as well as global resources to support such activities. These figures would facilitate the creation and adoption of locally relevant guidelines.
- The age group subclassification of the MENA reflects that the largest proportion of the population are children, teenagers, and young adults. This situation is very different from ageing populations in HICs, and provides an opportunity to prevent raised BP through population-based initiatives while considering a life-course strategy.^13^ This could include early education and awareness programmes, campaigns, and seminars to provide knowledge on hypertension, and educating healthcare staff on the importance of early identification and advocacy towards a healthier lifestyle to prevent hypertension. A strategy targeting children may be the most cost-effective approach, but the benefits may take decades to realize. In the short term, funding would be essential to support these interventions that are likely to have a much greater cost–benefit ratio when compared with dealing with the consequences of hypertension later in the life-course.
- With limited local funding to support the education of primary care clinicians, nurses, and other health workers, funding support from pharmaceutical and medical companies would be crucial to provide continuous medication education to ensure best practices are followed in hypertension care.
- Broadening population screening and awareness programmes (such as the MMM campaign) or assigning specific screening clinics in LMICs in the MENA region would be an important step to increase awareness and treatment. Ideally, healthcare providers should receive thorough training on hypertension management including refresher courses, with a focus on preventive cardiology.
- A call to action is urgently needed with specific steps to stop the early development of hypertension to curb the tsunami of poorly controlled BPs.

### 3.5 South and Central Asia

Countries: Afghanistan, Bangladesh, Bhutan, India, Kazakhstan, Kyrgyzstan, Maldives, Nepal, Pakistan, Sri Lanka, Tajikistan, Turkmenistan, Uzbekistan

#### 3.5.1 Epidemiology

With a population of almost two billion, the South and Central Asia Region (SACA) is the largest region under the ISH’s remit in terms of population size. The region consists of heterogeneous ethnicities and income groups, most of them falling under the upper-middle to low-middle-income groups.^144^ According to WHO, the estimated age-adjusted prevalence of hypertension is ∼27% (ranging from 25% in Bangladesh to 30% in Pakistan).^145^ This figure is also reflected in the 31.3% with hypertension in those participating in opportunistic screening as part of MMM (*Figure 4*). According to WHO, the awareness, treatment, and control rates vary substantially from 33 to 83%, 30 to 70%, and 7 to 30%, respectively. Kazakhstan has the highest awareness (male: 80%, female: 86%), treatment (men: 66%, women: 74%), and control rate (men: 25%, women: 34%), whereas Nepal has the lowest rates for awareness (men: 30%, women: 35%), treatment (men: 16%, women: 21%), and control (men: 5.9%, women: 8.5%). Overall women have a higher BP control rate compared with men in all countries in the region.^4^

#### 3.5.2 Challenges

Over the past three decades, the mortality rate due to hypertension in the SACA region has doubled.^146^ This population is burdened in terms of comorbidities, which comprise 70% of patients with two or more comorbidities.^147^

- It is estimated that ∼50% of hypertensive patients in the region do not adhere to medication.^148^ This is likely due to the huge gap between physician capacity and patient need for hypertension care as only physicians are allowed to prescribe antihypertensive medicines.^149^ Also, most of the countries in the region practice 1-month refilling for medication which may contribute to non-adherence.^150^
- Salt intake in the region is approximately double the WHO recommendation (<5 g/day), with a significant proportion of salt intake derived from salt added during cooking and discretionary use at the table.^151^ Although salt reduction initiatives have been proposed in several countries of the region, they are yet to be fully implemented and evaluated.
- The prevention and control of hypertension in the SACA region are complex and differ vastly among countries, with strong regional, economic, and cultural influences. It is essential to close the gap in differences in practice and delivery of healthcare.

#### 3.5.3 Opportunities

The region has the following opportunities to improve BP control:

- Strong collaboration between stakeholders,^6,13^ such as governmental and non-governmental organizations, are important to implement best practice strategies in preventing and managing hypertension. The Indian Hypertension Control Initiative (IMCI) supported by Resolve to Save Lives and implemented by the Government of India is an example of such a collaboration.^152^
- Improved detection and management of raised BP by using large population-based awareness and screening campaigns such as MMM. Many countries in SACA who participated showed positive results with over 850 000 people screened in 2018 and 2019 (*Figure 4*).^153^
- Task sharing with non-physician health workers. Several studies from the region consistently show that the engagement of non-physician workers such as nurses and community health workers was cost-effective and effective in reducing BP.^154–157^ New guidelines from the WHO and ISH highlight the importance of team-based care, but also treatment without laboratory investigations and self-measurement by patients in resource-poor settings.^25,158^ However, these guidelines are yet to be implemented in practice. The results of an ongoing study in Nepal [NCT04521582] where community health workers are prescribing antihypertensive medication could have an important policy implication for task-sharing in the region.
- Population-based salt reduction efforts, including potassium salt substitutes.
- Scaling-up of SPC therapies and multi-month prescription refills. Countries should develop country-specific strategies to include SPCs on country Essential Medicine Lists, aligned with the WHO that added fixed-dose combination antihypertension medication to their Essential Medicine List in 2019.^159^ As the South Asian population is at particularly high risk for CVD and metabolic diseases, clinical trials are needed to recommend relevant treatment approaches.^25^
- Implementing universal health coverage. To finance hypertension care, other countries in the region may learn from Sri Lanka and Bhutan, that provide free hypertension care as part of their universal health coverage,^160^ or alternatively should develop an equity-based health insurance model.
- Implementing the WHO HEARTS technical package.

### 3.6 Asia Pacific

Countries/regions: Australia, Brunei Darussalam, Cambodia, China, Cook Islands, Democratic People’s Republic of Korea (North Korea), East Timor, Fiji, Indonesia, Japan, Kiribati, Laos, Malaysia, Marshall Islands, Micronesia, Mongolia, Myanmar, Nauru, Niue, New Zealand, Palau, Papua New Guinea, Philippines, Republic of Korea (South Korea), Samoa, Singapore, Taiwan, Thailand, Taiwan, Vietnam

#### 3.6.1 Epidemiology

Asia is a diverse continent, and the prevalence of hypertension has increased over the last 30 years.^140^ BP levels *per se* have also increased in Asian countries, being among the highest in the world.^75^ Furthermore, BP control in Asia is relatively poor compared with Europe, Canada, and the USA (*Figure 1*). Even within the Asian region, there is large heterogeneity in the awareness, treatment, and control status of BP,^144,161^ partly due to relevant ethnic aspects and differences in diet, lifestyle, and sociodemographic factors.

The slope of the association between BP and cardiovascular events is steeper in Asians compared with Westerners.^162^ Data from the recent STEP (Strategy of Blood Pressure Intervention in the Elderly Hypertensive Patients) trial, conducted in China, showed that strict BP control (systolic BP 110–130 mmHg) was superior to standard systolic BP control (130–150 mmHg) for preventing cardiovascular events.^163^ This suggests that elderly Asians would benefit from strict BP control to reduce cardiovascular risk.

#### 3.6.2 Challenges

**Figure 6:**
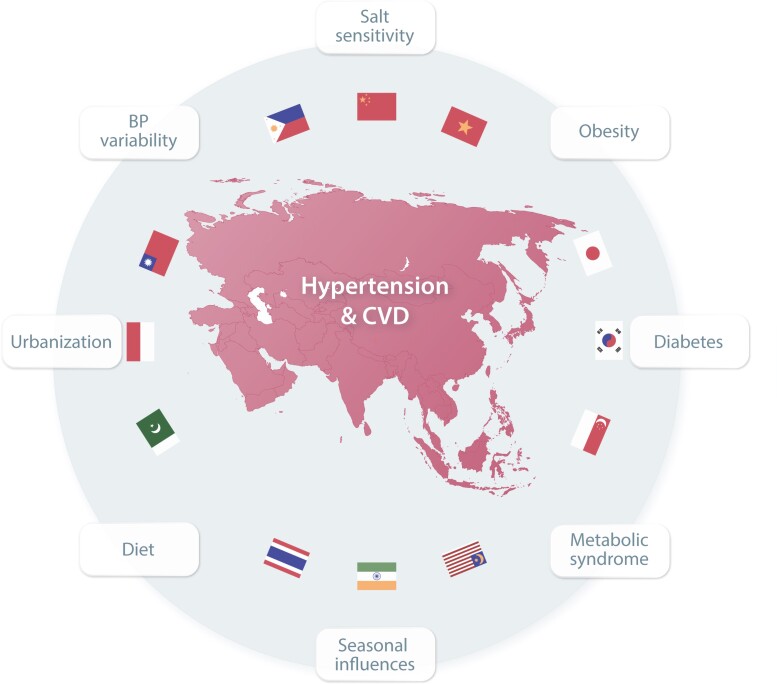
Factors contributing to hypertension and cardiovascular disease in Asia (adapted and modified from Kario *et al*.).^144^

Several factors contribute to the development of hypertension and CVD in Asia (*Figure 6*).^144^

- Higher salt intake and salt sensitivity: Asians are genetically predisposed to salt sensitivity.^162^ Salt intake is high compared with other populations,^164^ and exceeds the WHO recommendations (<5 g/day). For example, average salt intake is >10 g/day in Vietnam, China, Korea, Japan, and Thailand.^165^

Partial or complete substitution of salt with potassium chloride is an effective and simple way to reduce salt intake, and thereby BP and cardiovascular event rates.^91^ A digital therapeutic strategy designed to facilitate lifestyle modifications, including salt restriction and body weight reduction, successfully reduced home and ABPM in patients with hypertension.^162^

- Obesity: The body mass index threshold associated with an increased risk of prehypertension and hypertension is 25 kg/m^2^ in Asians compared with 30 kg/m^2^ for Westerners.^162^ Thus, body weight control, especially in younger and middle-aged adults, is important for Asians.
- Hypertension phenotype: Masked hypertension is more prevalent in Asian than in Western countries due to higher rates of abnormal patterns of BP variability, including an exaggerated early morning BP surge and non-dipper/riser phenotypes of nocturnal BP.^162^ Thus, identifying and treating masked hypertension phenotypes seem particularly important for cardiovascular risk reduction in Asian populations. Three prospective observational studies conducted in Asia (Ohasama, J-HOP, and HONEST) showed that morning hypertension detected by HBPM is associated with a higher risk of CVD, regardless of office BP.^162^ Another important feature of hypertension in Asian populations is high night-time BP, which is commonly associated with high salt sensitivity and salt intake. The presence of isolated nocturnal hypertension is a risk factor for organ damage and CVD even when office and/or morning BP are well controlled.^166^ Nocturnal hypertension is often found in high-risk patients with comorbidities such as diabetes, CKD, sleep apnoea, and risk of heart failure.^167^

#### 3.6.3 Opportunities to improve BP control

Several actions can be taken to improve hypertension management in Asia.

- Better awareness, screening, and monitoring: Screening for hypertension in public places and self-measurement of BP using HBPM at home and/or the work site may help increase awareness.^162,168^ Results from MMM in Asia for over 1 million individuals screened in 2018 and 2019 (*Figure 4*) showed collectively that 34% had BP in the hypertensive range, with an overall control rate of 32.7%. For 2019, MMM data indicate a prevalence of hypertension of 30.6% in East Asia and 47.8% in South-East Asia and Australasia; corresponding hypertension awareness rates were 59.0% and 66.5%.^153^ Of patients with hypertension in the Asia Pacific, only 16.8–28.6% were on antihypertensive medication. For treated patients, 33.4% of those in East Asia and 36.8% in South-East Asia and Australasia had uncontrolled BP.^153^ Telemedicine may represent a valuable approach to help deliver effective care to patients with hypertension.^162,169^

Development of HBPM devices capable of measuring nocturnal BP and other information and communication technology-based strategies are key developments in the widespread implementation of anticipation medicine strategies to detect and prevent cardiovascular events in patients with hypertension, particularly masked hypertension.^162^

- Increased uptake of telemonitoring: in the COVID-19 era, telemedicine has become a popular option to ensure patient and physician safety, and to facilitate infection control. Telemonitoring can be implemented more widely to improve access to care and patient outcomes. Wearable BP monitoring devices have great potential and are increasingly developed (although there are challenges to validate these devices for accuracy^170^),^162^ and an ABPM technology platform has great potential to facilitate diagnostic and treatment decisions without the need for an office visit.^169^
- Salt intake reduction programmes:^168^ Most successful programmes include multicomponent strategies and are aligned with the WHO recommendations.
  Asian countries have implemented many programmes to reduce population salt intake.^171^ A significant reduction in salt intake in both children and adults was achieved through the School-based Education Program to Reduce Salt Intake in Children and Their Families (School-EduSalt) in China.^172^ The ‘Tokyo Declaration in Promotion of Salt Reduction’ recommended six strategies to achieve a salt intake of <6 g/day.^173^ The Okinawa Declaration on the unity of hypertension societies in Asian countries and regions to overcome hypertension and hypertension-related diseases was announced in 2021.^174^ The impact of such programmes is shown by the National Health and Nutrition Survey of Japan, which showed small but gradual reductions in salt intake between 2005 and 2018, with a corresponding increase in the number of patients being treated with antihypertensives and decreases in average systolic BP.^56^ In parallel with improved BP control, there was a 17.5% decrease in stroke deaths.^56^
- Optimization of antihypertensive therapy: CCBs (sodium-independent BP-lowering effect),^161,162^ renin–angiotensin system inhibitors, and diuretics are effective in salt-sensitive hypertension. For strict BP control, SPCs are preferred, where feasible, as they promote drug adherence. Although traditional medicines are not recommended to treat hypertension due to a lack of evidence, a clinical trial has demonstrated the efficacy of a Chinese herbal formula (gastrodia-uncaria granules) in treating masked hypertension.^175^
- HOPE Asia Network: The HOPE Asia Network was set up to improve the management of hypertension in Asia with the goal to achieve ‘zero’ cardiovascular events in the region. This resulted in the publication of several consensus documents and recommendations covering almost all major topics relating to the management of hypertension.^161^ The HOPE Asia Network model provides a good example of the local interpretation, modification, and dissemination of international best practice to benefit specific populations in collaboration with local hypertension societies.

## 4. Actions to ensure evidence-based hypertension care for cardiovascular risk reduction

This section will discuss evidence-based strategies to improve hypertension care for reducing CVD and eliminate related disparities among and within countries. To recommend such actions, a thorough understanding of global disparities in care is required.

### 4.1 Reasons for global disparities in hypertension care among and within countries

The reasons for the global disparities in hypertension care are multifactorial and affect the populations at a different level in both LMICs and HICs. A major challenge has been the overall public health emphasis on managing infectious diseases with inadequate focus on the detection and management of hypertension. The key factors are listed below and summarized in *Table 5*.

**Table 5:** Factors associated with disparities in hypertension care globally

	Low–middle-income countries	High-income countries
Social determinants	Poverty Food insecurity Maternal malnutrition Poor nutrition during periconception Low education, unemployment, and health illiteracy Racism Effects of wars and political conflicts	Neighbourhood poverty, low education, poor social support, unemployment in racial/ethnic, low-income groups, and migrants Institutional racism in policies and practices at multiple levels
Environmental determinants and commercial determinants	Poorly planned urbanization and unregulated trade policies promoting unhealthy behaviours in the vast majority of the population Tobacco Calorie-dense diets High salt diets sugar-sweetened beverages low fruit and vegetable consumption, high trans-fats and saturated fat intake, high alcohol consumption Physical inactivity Loss of green space Air pollution	Unhealthy behaviours promoted by social deprivation (listed above) specific to disadvantaged groups (low-income families, ethnic/racial subgroups): high salt diets, sugar-sweetened beverages low fruit and vegetable consumption, high trans-fats and saturated fat intake, high alcohol consumption sugar-sweetened beverages physical inactivity, prolonged sitting time, and screentime Loss of green space Air pollution
Health systems	Grossly under-funded/non-existent public health for hypertension care. Lack of implementable standardized clinical care for management of hypertension Lack of social insurance for hypertension care Out-of-pocket expenditure for antihypertensive medications and poor adherence Shortage of qualified physicians and nurses Knowledge gaps in hypertension care Poor access to quality hypertension care in most countries Lack of social insurance coverage for treatments that have better adherence, e.g. single-pill combination antihypertensive medications	Under-funded public health for hypertension care in some settings (e.g. the USA) Gaps in some practices especially physician inertia to intensity treatment in patients with comorbidities Clinician–patient communication barriers with racial/ethnic sub-groups Variable access to quality hypertension care for disadvantaged groups Insufficient social insurance coverage for highly efficacious treatments in some settings, e.g. single-pill combination Antihypertensive medications, and more intensive BP monitoring (e.g. 24 h ambulatory and home monitoring) Non-adherence to antihypertensive treatment

#### 4.1.1 Social and environmental determinants during the life-course

Populations exposed to poverty, food insecurity, low education and health illiteracy, high rates of maternal malnutrition, and poor nutrition in the periconception period with accelerated post-infancy weight gain are particularly susceptible to hypertension and related CVD later in life.^176^ In addition, unplanned urbanization and poor trade policies promote unhealthy behaviours such as poor diet (high salt and low fruit and vegetable intake, high saturated and trans-fats), physical inactivity, tobacco and alcohol use, and obesity fuel the rise in BP over the lifespan. Pollution (air, water, noise, and light),^177^ psychosocial stress, and a loss of green space are emerging risk factors of hypertension.^6^ The long-term implications of climate change for both HIC and LMIC should not be underestimated by clinicians and policymakers, where extreme temperatures,^178,179^ flooding, and drinking water salinity may all impact BP and its control. Furthermore, people living in conflict zones (e.g. Afghanistan, Iraq, Libya, Palestine, Syria) or exposed to stressful conditions may have a higher risk of hypertension and CVD.^142^

#### 4.1.2 Health systems

Despite the demonstrated benefits of effective drug treatment and the existence of several international clinical practice guidelines, hypertension care indicators (awareness, treatment, and control rates) remain poor in many LMICs. The health systems performance in achieving better hypertension care correlates positively with a country’s economic development. From that perspective, countries in Latin America and the Caribbean (Brazil, Costa Rica, Ecuador, and Peru) have performed better relative to their gross domestic product (GDP) per capita whereas countries in SSA performed worst.^74^ In many countries in South Asia and Africa, healthcare is often sought through the poorly regulated private or informal sector. The rural areas have a shortage of qualified physicians, and nurses. Moreover, serious gaps exist in the knowledge and practices of providers regarding the management of hypertension.^180,181^ For example, sedatives are used to treat hypertension by up to one-third of providers in Pakistan, and antihypertensive medications are often stopped once BP is controlled.^180^

Donor-assisted funding for NCDs has been poor, with a budget discrepancy of 10:1 in favour of infectious diseases. Thus, local governments have not prioritized NCD prevention and control. For example, in South Asia, Africa, Vietnam, and Malaysia non-physician health workers offer immunization, family planning, and maternal and preventive child-care services; they do not have the training or mandate to deliver hypertension care.

In some countries, drug procurement is a significant challenge, even for the essential class of first-line antihypertensive drugs, such as ACE-I, not available in at least 16 LMICs.^181,182^ The lack of standardized clinical care algorithms that are implementable in the local context with wide variations in drugs used, doses, and brand names complicates individual treatment and makes bulk purchasing and supply chain logistics extremely difficult.

Antihypertensive medications remain an out-of-pocket expense for many low-income families without social insurance, especially in Africa and South Asia.^181^ Poor adherence due to the cost of drugs is a significant patient-related barrier to BP control.^182^ Non-adherence is also one of the leading drivers of suboptimal BP control in HIC, although the main causes for non-adherence (i.e. polypharmacy)^183^ are possibly different to those in LMIC. Moreover, patient-related barriers such as the cost of travel to the clinics and the opportunity time of daily wage workers could impede access, especially for men who are less likely to be treated for hypertension than women.^184^ There is little evidence on the cost-effectiveness of screening for hypertension in these LMICs settings unless accompanied with health systems strengthening for treating hypertension and ensuring access to medications.^185^ Thus, outreach efforts with non-traditional models of hypertension care delivery at home or worksite need to be considered.^156,186^

Multiple studies have shown that most patients with hypertension, especially those with obesity or comorbidities, require more than one drug to control BP.^187,188^

SPC drugs with flexible doses of two antihypertensive medications, many of which are off-patent and hence relatively low cost, improve adherence and possibly BP control. Major international hypertension management guidelines recommend SPCs.^25,187,189^ However, in most LMICs, different SPCs are not available and are not subsidized. Moreover, the widespread use of SPC drugs requires a robust healthcare delivery infrastructure which does not exist in many LMICs.^190^

#### 4.1.3 Race/ethnic-based and social inequities in policies and practices related to hypertension and CVD

The racial/ethnic inequities in hypertension and CVD relate primarily to social and economic factors such as neighbourhood poverty, low education, poor social support, unemployment, being uninsured, lack of affordability, psychosocial stressors due to racism and with unhealthy behaviours and poor adherence to medications. Moreover, almost 30 million people from low-income families in the USA are still without any form of health insurance and Blacks and Hispanics are 1.5 and 2.5 times, respectively, more likely to be uninsured than Whites, and therefore with poor access to hypertension care.^188^

These disparities were further unmasked during the COVID-19 pandemic, which affects people with hypertension-related CVD preferentially and led to disproportionately more deaths in Black, Latino, South Asian, and other populations in the USA, UK, Europe, and Canada, attributed in part to institutional or structural racism.^98,191–193^ The latter refers to practices and policies of institutions, workplaces, and health systems that chronically place certain racial/ethnic groups at a disadvantage.^193^ The adverse experiences, even if perceived, are associated with psychosocial stress, mistrust in healthcare, suboptimal adherence to antihypertensive medications, and poor outcomes.^188^

### 4.2 Way forward and call to action

Immediate action is needed to address suboptimal hypertension care globally and related disparities. The WHO’s Global Action Plan for Prevention and Control of NCDs aims to reduce the prevalence of hypertension by 25% by 2025, relative to 2010.^138^ Subsequently, the United Nations’ Sustainable Development Goal (SGD) 3.4 aims to reduce premature CVD mortality by 30% by 2030; relative to 2015.^194^ Improving BP control by 50% is central to achieving this goal.^194^ However, many LMICs have not observed any improvement in hypertension burden or BP control (*Figure 1*).^4^ In HIC, gaps in hypertension control and inequities persist, which need to be bridged. It is possible to achieve prevention and control of hypertension through multifaceted, cost-effective efforts, including population-level policy initiatives and health systems strategies targeting high-risk individuals. For example, a recent modelling study showed that three essential public health interventions, including reducing sodium intake by 30%, eliminating the intake of artificial trans-fatty acid, and improving coverage of antihypertensive drugs to 70% could save millions of lives by preventing NCDs.^195^

**Figure 7:**
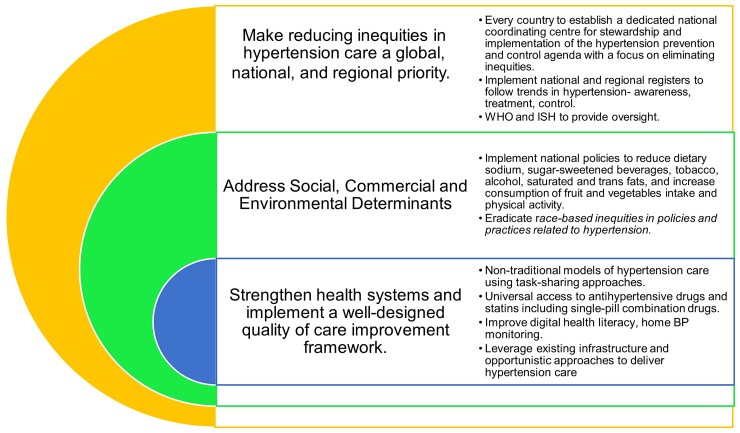
Key recommendations for eliminating global inequities in hypertension care.

We recommend the following actions for improving BP awareness, treatment, and control rates by at least 30% by 2030, relative to 2015, for CVD risk reduction, and a particular focus on eliminating related disparities (*Figure 7*).

1.ȃMake reduction in inequities in hypertension care a global, regional, national priority.

We propose a Global Hypertension Care Task Force jointly led by WHO, ISH, and other professional societies. The Task Force would facilitate policy dialogues and measures, including technical assistance to catalyse improvement in BP control rates. In addition, every country should establish a dedicated national coordinating centre for stewardship and implementation of the hypertension prevention, detection, and control agenda with effective outreach to the disadvantaged populations at high risk of hypertension and national surveillance and reporting of standardized hypertension care indicators. The centre would also facilitate policy dialogues with the governments, set targets for priority programmes and policies according to the local context, and advocate for large-scale implementation of well-funded programmes to eliminate inequities in hypertension care.

2.ȃImplement national policies and interventions to reduce dietary salt, sugar-sweetened beverages (SSB), obesity, tobacco, alcohol, saturated and trans-fats, and increase consumption of fruit and vegetables intake and physical activity. Support the eradication of social and institutional racism.

Lifestyle modification is essential and less costly for the prevention and non-pharmacological treatment of hypertension. Policy strategies for hypertension prevention require multi-sectoral processes, including taxation and subsidies, marketing healthy foods, and improving the built environment to promote physical activity.

- Dietary salt: In patients with hypertension, a high salt intake is associated with a higher risk of CVD and death.^91,196,197^ The WHO has designated a reduction in salt intake to <5 g/day as a ‘best buy’, i.e. one of the most cost-effective and affordable interventions to avoid premature deaths and reduce the economic impact of NCDs. In HICs, the primary source of dietary salt is via packaged foods and food and sauces prepared outside the home. Policy-level interventions, especially food product re-formulation, have been successful in HICs, for example, in Finland.^198^ About 75 countries have a national policy to reformulate food to lower dietary salt, albeit implementation is at different stages. However, in LMICs, more than half of dietary salt is from discretionary sources added while cooking or at the table.^199^ Of note, African and Asian populations may have more salt-sensitive hypertension, and average salt intake is higher in Asian countries (e.g. >12 g/day in China).^165^ As of 2015, almost half of the 1.13 billion people with hypertension lived in South Asia or East Asia.^2^ The Salt Substitute and Stroke Study (SSaSS) study conducted in rural China demonstrated the benefit of substituting traditional salt with potassium salt on BP and CVD event rates.^91^ Not only did this intervention reduce sodium intake, but it addressed suboptimal potassium intake as experienced in high-^200^ and low^201^-resource settings, with higher potassium contributing to BP lowering.
- Tobacco control: Although not causally related to high BP, tobacco control is one of the most cost-effective legislative strategies for CVD risk reduction in the general population and for those with and at risk of hypertension.^202^ Although 168 countries have ratified the WHO’s Framework Convention Tobacco Control (FCTC), more than 40% of LMICs do not ban advertising cigarette advertising.^203^
- Trans-fats, alcohol, sugar, and SSB consumption: Reducing trans-fat consumption is expected to lower CVD mortality in patients with hypertension.^204^ The primary source of trans-fat is partially hydrogenated vegetable oils which are cheap to produce and have a long shelf life, and therefore more profitable for the food industry.^205^ WHO recommends a complete ban on trans-fats, or a maximum limit of 2% trans-fat in all foods. Likewise, avoiding binge drinking and reducing alcohol to less than two daily drinks lower BP and reduces deaths. Increasing taxes and prices reduces alcohol consumption.^206^ Sugar and SSB taxes are projected to have substantial health gains.^207^ Over 40 countries, including Mexico, South Africa, the Philippines, and India, have implemented taxation on SSB, projected to reduce obesity and associated elevated BP.^208^
- Fruit and vegetable intake: Based on the results of the Dietary Approaches to Stop Hypertension (DASH) trial, a diet rich in fruit and vegetable is recommended for lowering BP.^37^ However, the cost of one serving of vegetables and fruits relative to income per household member is several-fold higher in LMICs than in HICs, respectively.^37,209,210^ Trade policies and subsidies must protect the affordability of the seasonal produce for the local populations, with government subsidies for the marginalized high-risk racial and low-income groups.
- Physical activity and school-based nutritional programmes: Systematic reviews of RCTs on school-based lifestyle interventions, including increasing physical activity, show a beneficial impact on BMI and BP.^211^ Parental involvement augmented the beneficial effects of interventions.^211^ Policies to ensure safe urban built environments will not only promote easier physical activity in the youth, but across the whole population.^13^
- Eliminate racial and social injustice in hypertension care: Finally, racism needs to be viewed as a social problem, not a biological construct.^10^ Proactive anti-racism measures are required to ensure health equity in hypertension care through advocacy, policies, and practices that proactively engage ethnic/racial groups and low-income families. Such measures include contextually tailoring interventions to ensure effective hypertension care delivery to disadvantaged populations.^10^

3.ȃStrengthen health systems and implement a well-designed quality-of-care improvement framework and ensure outreach to specific racial/ethnic groups and low-income populations that is sustainable and cost-effective.

Strengthening health systems using a well-designed quality improvement framework that addresses multiple barriers to hypertension care is essential. In addition, such efforts must ensure outreach to the disadvantaged populations, including low-income and racial/ethnic groups, and be cost-effective, acceptable, affordable, and sustainable.

The following evidence-based health systems interventions are likely to be most impactful in reducing the burden of uncontrolled hypertension and CVD, and related disparities:

(a)ȃScaling-up evidence-based, non-traditional models of hypertension care with task-sharing tailored to the local setting.

Several studies and systematic reviews of randomized trials evaluating health systems strategies have shown that single interventions such as training physicians alone, or patient education alone, have modest to no benefit on BP control.^212^ However, combined interventions including team-based approaches addressing multiple barriers to BP control are likely to yield clinically meaningful BP lowering and CVD risk reduction.^212–214^

The Control of Blood Pressure and Risk Attenuation-Bangladesh, Pakistan, Sri Lanka (COBRA-BPS) trial evaluated a multicomponent intervention including the non-traditional model of trained community health worker-led HBPM, home health education underscoring lifestyle modifications and adherence to antihypertensive medications, and trained physicians tailored to the local public health care infrastructure. The intervention improved BP control by 22% over 2 years and was cost-effective and affordable (less than US$2 per capita annually).^154,156^ The intervention also improved antihypertensive medication intensification, and some aspects of quality of life. Likewise, several studies have shown benefit of task-sharing approaches with community health workers delivering hypertension care via BP monitoring, lifestyle counselling, and linkage to clinics in urban communities in Argentina, India, Nepal, Pakistan, and Kenya.^104,155,215,216^ More recently, the benefit of a community health worker-led multifaceted intervention on BP control has also been demonstrated in rural villages in China.^217^

Non-traditional models of care are effective in racial minorities in HICs as well. In the USA, pharmacist-led, barbershop-based hypertension care has been shown to successfully lower BP in high-risk Blacks with hypertension relative to usual care,^112^ albeit sustainability and cost-effectiveness of the approach remain to be established.^112^

Thus, scaling-up non-traditional models of care tailored for disadvantaged populations will likely reduce disparities in BP control and CVD substantially.

(b)ȃEnsure universal access to antihypertensive medications including initial SPC therapy:

Antihypertensive medications must be available at no or subsidized cost to patients in primary healthcare centres. Universal health coverage, including access to quality and affordable essential medicines for all, is advocated by United Nations’ Sustainable Development Goals.^194^ Adherence to antihypertensive treatment must be promoted through both scheduled and opportunistic interactions between patients and healthcare professionals given the potential positive impact of better adherence on BP control in individual patients^218^ and healthcare systems.^219^ There are new direct objective diagnostic approaches to detecting, monitoring and management of non-adherence to antihypertensive treatment including self-HBPM but they are currently only routinely available in some HICs. More research is required to develop affordable and scalable diagnostic methods and therapeutic interventions for non-adherence to antihypertensive treatment in LMICs.

Health insurance reforms must include universal coverage for hypertension care, including non-traditional models integrated into primary care, and reduce out-of-pocket expenditure for antihypertensive drugs. Some examples of quality access for hypertension care include health systems in Canada, and Scandinavian countries, albeit racial/ethnic disparities need improvement. In addition, the hypertension quality improvement programme with SPC antihypertensive medications in the Kaiser Permanente network in California showed marked improvement in BP control.^113^ Such models could be adapted in Central and Eastern Europe, North Africa, and the Middle East to bridge the gap between high BP awareness and control rates.

(c)ȃLeverage existing infrastructure and opportunistic approaches to deliver hypertension care.

Leveraging the existing platform offers an opportunity to achieve population-level prevention of hypertension at marginal costs.^220^ The partnership between WHO’s Global Hearts Initiative with Resolve to Save Lives to produce the HEARTS Technical Package provides essential modules for training health providers.^221^ The partnership between WHO and Resolve to Save Lives has shown promising results with an additional 3 million people on hypertension treatment. The impact of this programme on improving BP control needs evaluation.

Opportunistic screening, awareness, and treatment of hypertension should also be encouraged by leveraging existing infrastructure for maternal and child health services, and infectious diseases (e.g. COVID-19, HIV, tuberculosis), public–private partnerships should be encouraged (e.g. worksite) to provide for unmet needs in special populations.^220^ For example, delivery of hypertension care could leverage the HIV care infrastructure funded by the US President Emergency Preparedness Funds for Aids Relief (PEPFAR) in SSA, where hypertension awareness is poor, while the burden is rising steeply.

(d)ȃImprove digital health literacy for future innovations in digital health, HBPM.

Evidence is accumulating on the potential benefit of digital health interventions for hypertension care. For example, smartphone use by the health workers and patients for virtual follow-ups improves adherence to antihypertensive medications and linkage to clinics.^222^ More than 80% of the population of LMICs have mobile phones, and a large majority of villages are connected with mobile technology.^215^ Telemonitoring is especially valuable during the social distancing requirements imposed by the COVID-19 pandemic with dropping clinic attendance. However, there are concerns regarding the potential widening of disparities racial and ethnic populations may not be tech-savvy.^98^ Therefore, measures to enhance digital literacy and additional solutions are needed for regions and people where information technology is still under-deployed or underused.

The use of home BP monitors improves treatment adherence and BP control when coupled with other interventions and need to be used more widely.^223^ ABPM (24 h, daytime, or night-time) is a better predictor of long-term CV outcomes than clinic BP^224^ and is recommended by the Canadian Hypertension Education Program in 2005 and NICE guidelines in 2011 for diagnosis of hypertension. However, the feasibility and cost-effectiveness of scaling-up ABPM relative to office BP or HBPM, particularly for repeated use in the long-term management of hypertension, remain to be studied in most settings including in HICs.^10^

## 5. Conclusion

Based on robust empirical data and modelling studies, progressive implementation of affordable, and equitable hypertension care between 2020 and 2030 could save the lives of more than 100 million people aged 30–70 years, who would otherwise die prematurely, and help achieve the Sustainable Development Goal 3.4.^195^ Such efforts would also reduce the hypertension-related disability burden on all health systems. Therefore, WHO and country leadership should prioritize improving the implementation of national programmes for hypertension care with outreach to the marginalized communities. They must also facilitate robust accountability and monitoring to achieve the targets and institutionalize establishing a national registry and surveillance on hypertension.

Additional research should be encouraged on novel therapies and rapidly scalable, low-cost interventions for eliminating inequities in hypertension care. These studies must also record patient-reported outcomes. Major donors, including the World Bank and Gates Foundation, need to invest in large programmatic initiatives to fight hypertension—the world’s biggest silent killer.

## Supplementary material

Supplementary material is available at *Cardiovascular Research* online.

## Authors’ contributions

A.E.S., B.W., and M.T. conceptualized the design and paper outline; N.R.P., A.D., N.A.K., P.M.N., J.A., D.N., K.K., and T.H.J. led the different sections. The following authors contributed to drafting the different sections: Lessons learnt (G.S., R.M.T., T.J.G., H.I., N.R.P.); SSA (A.D., E.S.W.J., A.O.); Americas (N.A.K., C.S.M., B.M.W., D.B., R.D.W.); Europe (P.M.N., S.B., U.M.S., T.U.); MENA (J.A., G.H., A.S.); SACA (D.N., M.P., U.R., M.I.); Asia Pacific (K.K., J.-G.W., M.P.S., C.S.M., P.K.); Action for cardiovascular risk reduction (T.H.J., H.B., F.J.C., E.M., T.K.). All authors drafted and reviewed the manuscript critically and gave final approval of the paper to be published.

**Collaborators (internal document reviewers):** George Bakris (USA); Norm Campbell (Canada); Thomas Beaney (UK); Claudio Borghi (Italy); Lizzy Brewster (Netherlands); Lebo Gafane-Matemane (South Africa); Krasimira Hristova (Bulgaria); Alexandra Konradi (Russia); Reinhold Kreutz (Germany); Michael Hecht Olsen (Denmark); Raj Padwal (Canada); Priscilla Prestes (Australia); Nicolás Renna (Argentina).

## Supplementary Material

cvac130_Supplementary_Data

## Data Availability

Since this paper is a review paper there is no data to be made available.
