# Supplementary material for: Addressing global disparities in blood pressure control: perspectives of the International Society of Hypertension
Source: Cardiovasc Res. 2022 Oct 11;119(2):381–409. doi: 10.1093/cvr/cvac130 (PMC9619669; doi:10.1093/cvr/cvac130)
Supplement: cvac130_Supplementary_Data [file cvac130_supplementary_data.zip › ISH Global Review_Suppl_R1.docx]

**Addressing global disparities in blood pressure control: perspectives of the International Society of Hypertension**

**Short title**: Addressing global disparities in BP control

Aletta E Schutte^1^, Tazeen H Jafar*^2^, Neil R Poulter*^3^, Albertino Damasceno*^4^, Nadia Khan*^5^, Peter M Nilsson*^6^, Jafar Alsaid*^7^, Dinesh Neupane*^8^, Kazuomi Kario*^9^, Hind Beheiry^10^, Sofie Brouwers^11^, Dylan Burger^12^, Fadi Charchar^13^, Myeong-Chan Cho^14^, Tomasz Guzik^15^, Ghazi Haji^16^, Muhammad Ishaq^17^, Hiroshi Itoh^18^, Erika Jones^19^, Taskeen Khan^20^, Yoshihiro Kokubo^21^, Praew Kotruchin^22^, Elizabeth Muxfeldt^23^, Augustine Odili^24^, Mansi Patil^25^, Udaya Ralapanawa^26^, Cesar Romero^27^, Markus Schlaich^28^, Abdulla Shebab^29^, Ching Siew Mooi^30^, U. Muscha Steckelings^31^, George Stergiou^32^, Rhian Touyz^33^, Thomas Unger^34^, Richard Wainford^35^, Ji-Guang Wang^36^, Bryan Williams^37^, Brandi Wynne^38^, Maciej Tomaszewski^39^

*Authors contributed equally.

1. School of Population Health, University of New South Wales, The George Institute for Global Health, Sydney, NSW, Australia; Hypertension in Africa Research Team, SAMRC Unit for Hypertension and Cardiovascular Disease, North-West University, and SAMRC Developmental Pathways for Health Research Unit, School of Clinical Medicine, University of the Witwatersrand, Johannesburg, South Africa
2. Program in Health Services and Systems Research, Duke-NUS Medical School, Department of Renal Medicine, Singapore General Hospital, Singapore, Duke Global Health Institute, Durham, Duke University, NC, USA
3. Imperial Clinical Trials Unit, School of Public Health, Imperial College London, London, W12 7RH, UK
4. Faculty of Medicine, Eduardo Mondlane University, Maputo, Mozambique
5. Department of Medicine, University of British Columbia, Canada; Center for Health Evaluation and Outcomes Sciences, Vancouver, Canada
6. Lund University, Department of Clinical Sciences, Skane University Hospital, Malmö, Sweden
7. Ochsner Health system. New Orleans, USA. Nephrology Consultant. Assistant professor Queensland University, Australia. Chair of Middle East and North Africa Advisory Group, International Society of Hypertension
8. Department of International Health, Johns Hopkins Bloomberg School of Public Health, Johns Hopkins University, MD, USA
9. Division of Cardiovascular Medicine, Department of Medicine, Jichi Medical University School of Medicine, Tochigi, Japan
10. International University of Africa, Sudan
11. Department of Cardiology, Cardiovascular Center Aalst, OLV Clinic Aalst, Aalst, Belgium; Department of Experimental Pharmacology, Faculty of Medicine and Pharmacy, Vrije Universiteit Brussel, Brussels, Belgium
12. Kidney Research Centre, Ottawa Hospital Research Institute, Department of Cellular and Molecular Medicine, University of Ottawa, Ottawa, Canada
13. Health Innovation and Transformation Center at Federation University, Ballarat, Australia
14. Department of Internal Medicine, College of Medicine, Chungbuk National University Cheongju, Korea
15. Institute of Cardiovascular and Medical Sciences, University of Glasgow, Scotland
16. Baghdad College of Medicine, Iraq
17. Pakistan Hypertension League, Karachi, Pakistan
18. Department of Endocrinology, Metabolism and Nephrology, School of Medicine, Keio University, Tokyo 160-8585, Japan
19. Kidney and Hypertension Research Unit, University of Cape Town, Cape Town, South Africa
20. World Health Organization WHO, Genève, Switzerland
21. Department of Preventive Cardiology, National Cerebral and Cardiovascular Center, Osaka, Japan
22. Department of Emergency Medicine, Faculty of Medicine, Khon Kaen University, Khon Kaen, Thailand
23. University Hospital Clementino Fraga Filho, Hypertension Program, Universidade Federal do Rio de Janeiro, Brazil
24. Circulatory Health Research Laboratory, College of Health Sciences, University of Abuja, Abuja, Nigeria
25. Department of Nutrition and Dietetics, Asha Kiran JHC Hospital, Chinchwad, India
26. Faculty of Medicine, University of Peradeniya, Sri Lanka
27. Renal Division, Department of Internal Medicine, Emory University School of Medicine, Atlanta, GA, USA
28. Dobney Hypertension Centre, School of Medicine - Royal Perth Hospital Unit and RPH Research Foundation, The University of Western Australia, Perth, Australia; Department of Cardiology and Nephrology, Royal Perth Hospital, Perth, Australia
29. College of Medicine and Health Sciences, United Arab Emirates University, Al Ain, United Arab Emirates
30. Department of Family medicine, Faculty of Medicine and Health Science, Universiti Putra Malaysia
31. Department of Cardiovascular & Renal Research, Institute of Molecular Medicine. University of Southern Denmark, Odense, Denmark
32. Hypertension Centre STRIDE‐7, School of Medicine, Third Department of Medicine, Sotiria Hospital, National and Kapodistrian University of Athens, Athens, Greece
33. Research Institute of the McGill University Health Centre, McGill University, Montreal, Quebec, Canada
34. CARIM - Cardiovascular Research Institute, Maastricht University, Maastricht, The Netherlands
35. Department of Pharmacology & Experimental Therapeutics and the Whitaker, Cardiovascular Institute, Boston University School of Medicine, Boston, MA, USA
36. Department of Hypertension, Centre for Epidemiological Studies and Clinical Trials, The Shanghai Institute of Hypertension, Shanghai Key Laboratory of Hypertension, Ruijin Hospital, Shanghai Jiaotong University School of Medicine, Shanghai, China
37. University College London (UCL), Institute of Cardiovascular Science, National Institute for Health Research (NIHR), UCL Hospitals Biomedical Research Centre, London, UK
38. Department of Internal Medicine, Division of Nephrology & Hypertension, Department of Nutrition and Integrative Physiology, Immunology, Inflammation and Infection Disease Initiative, University of Utah, Salt Lake City, UT, USA
39. Division of Cardiovascular Sciences, Faculty of Medicine, Biology and Health, University of Manchester; Manchester Heart Centre and Manchester Academic Health Science Centre, Manchester University NHS Foundation Trust, Manchester, UK

**Corresponding authors:**

Aletta E Schutte, University of New South Wales, Sydney, Australia

Tel. +61 450 315 918; Email: [a.schutte@unsw.edu.au](mailto:a.schutte@unsw.edu.au)

Maciej Tomaszewski, University of Manchester, United Kingdom

Tel. +44 161 275 0232; Email: [maciej.tomaszewski@manchester.ac.uk](mailto:maciej.tomaszewski@manchester.ac.uk)

**Supplementary Methods**

Estimates to create figures based on the May Measurement Month (MMM) data, were pooled data from MMM 2018 and 2019. Hypertension was defined as having a systolic BP ≥ 140 mmHg or diastolic BP ≥ 90 mmHg (based on the mean of the second and third reading) or being on anti-hypertensive medication. Where the second or third BP reading was missing, multiple imputation using chained equations was used to impute the mean, using an approach described previously based on the global data.^1, 2^ Mean systolic and diastolic BP levels were standardised according to the WHO world-standard population and assuming an equal male:female ratio. For national level awareness estimates, the proportion of participants with hypertension, and proportion aware were calculated for those with data recorded for awareness only, and for countries with at least 500 non-missing entries for awareness.

**References**

1. Beaney T, Burrell LM, Castillo RR, Charchar FJ, Cro S, Damasceno A, Kruger R, Nilsson PM, Prabhakaran D, Ramirez AJ, Schlaich MP, Schutte AE, Tomaszewski M, Touyz R, Wang JG, Weber MA, Poulter NR. May Measurement Month 2018: a pragmatic global screening campaign to raise awareness of blood pressure by the International Society of Hypertension. *Eur Heart J* 2019;**40**:2006-2017.

2. Beaney T, Schutte AE, Stergiou GS, Borghi C, Burger D, Charchar F, Cro S, Diaz A, Damasceno A, Espeche W, Jose AP, Khan N, Kokubo Y, Maheshwari A, Marin MJ, More A, Neupane D, Nilsson P, Patil M, Prabhakaran D, Ramirez A, Rodriguez P, Schlaich M, Steckelings UM, Tomaszewski M, Unger T, Wainford R, Wang J, Williams B, Poulter NR. May Measurement Month 2019: The Global Blood Pressure Screening Campaign of the International Society of Hypertension. *Hypertension* 2020;**76**:333-341.
